# How Has Molecular Biology Enhanced Our Undertaking of axSpA and Its Management

**DOI:** 10.1007/s11926-022-01092-4

**Published:** 2022-10-29

**Authors:** Mauro Fatica, Arianna D’Antonio, Lucia Novelli, Paola Triggianese, Paola Conigliaro, Elisabetta Greco, Alberto Bergamini, Carlo Perricone, Maria Sole Chimenti

**Affiliations:** 1 Rheumatology, Allergology and Clinical Immunology, Department of Systems Medicine, University of Rome Tor Vergata, Rome, Italy; 2 UniCamillus, Saint Camillus International University of Health Sciences, Rome, Italy; 3 Rheumatology, Department of Medicine, University of Perugia, Perugia, Italy

**Keywords:** Spondyloarthritis, Axial spondyloarthritis, Genetic, Immune response, Bone metabolism, Treatment target

## Abstract

**Purpose:**

This review aims at investigating pathophysiological mechanisms in spondyloarthritis (SpA). Analysis of genetic factors, immunological pathways, and abnormalities of bone metabolism lay the foundations for a better understanding of development of the axial clinical manifestations in patients, allowing physician to choose the most appropriate therapeutic strategy in a more targeted manner.

**Recent Findings:**

In addition to the contribution of MHC system, findings emerged about the role of non-HLA genes (as ERAP1 and 2, whose inhibition could represent a new therapeutic approach) and of epigenetic mechanisms that regulate the expression of genes involved in SpA pathogenesis. Increasing evidence of bone metabolism abnormalities secondary to the activation of immunological pathways suggests the development of various bone anomalies that are present in axSpA patients.

**Summary:**

SpA are a group of inflammatory diseases with a multifactorial origin, whose pathogenesis is linked to the genetic predisposition, the action of environmental risk factors, and the activation of immune response. It is now well known how bone metabolism leads to long-term structural damage via increased bone turnover, bone loss and osteoporosis, osteitis, erosions, osteosclerosis, and osteoproliferation. These effects can exist in the same patient over time or even simultaneously. Evidence suggests a cross relationship among innate immunity, autoimmunity, and bone remodeling in SpA, making treatment approach a challenge for rheumatologists. Specifically, treatment targets are consistently increasing as new drugs are upcoming. Both biological and targeted synthetic drugs are promising in terms of their efficacy and safety profile in patients affected by SpA.

## Introduction

Spondyloarthritis (SpA) encompasses a group of inflammatory diseases with a multifactorial origin, whose pathogenesis is linked to the genetic predisposition, the action of environmental risk factors, and the activation of immune response. Ankylosing spondylitis (AS) is certainly the most well-renown disease of the axial SpA (axSpA) spectrum and is characterized by the presence of typical radiological signs, included in the modified New York AS criteria [1]. On the other hand, the more recent ASAS (Assessment of Spondyloarthritis International Society) classification criteria for axSpA [2] can capture patients without radiographic signs of the disease termed non-radiographic axSpA (nr-axSpA) [3].

**Fig. 1:**
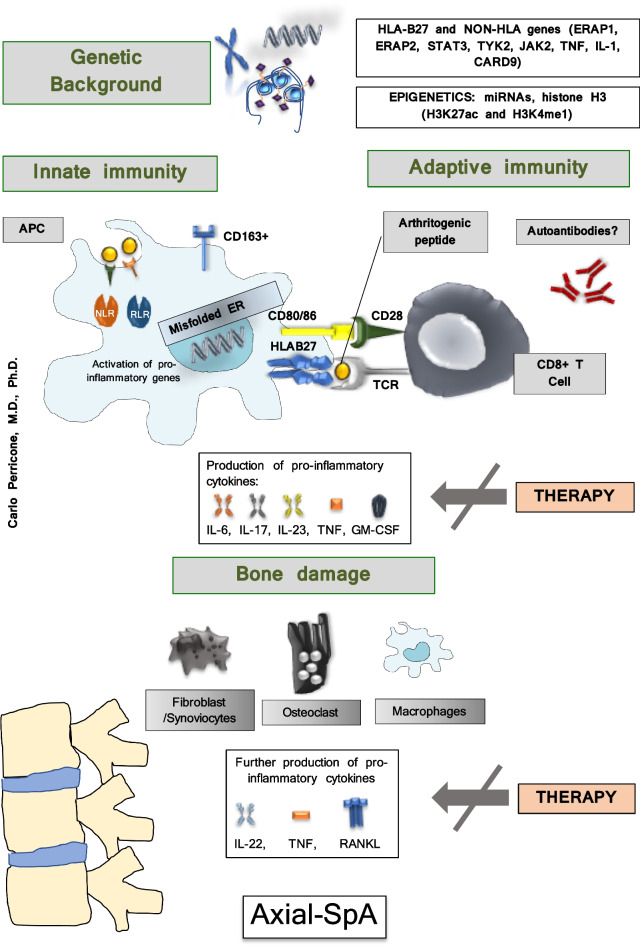
Pathogenesis of axSpA

A great deal of progress has been made in the knowledge of the pathogenesis of SpA. The cooperation between the various risk factors determines the activation of various inflammation cells with the release of a series of proinflammatory cytokines that lead to the development of various clinical manifestations [4]. After the discovery of HLA-B27, research has focused on other genetic factors, as well as immunological mechanism, recognizing a crucial involvement of innate and adaptive immune systems and a dysfunction of immune cells. The immune system, composed by various cells and released mediators, controls immune response and inflammation and chronic inflammation when its regulation and homeostasis are disrupted [5]. It has been known for years that in SpA, there is an alteration of bone metabolism which leads to long-term structural damage due to increased bone turnover, bone loss and osteoporosis, osteitis, erosions, osteosclerosis, and osteoproliferation. These effects can exist in the same patient over time or even simultaneously [6]. The pathogenesis of axSpA is summarized in Fig. 1. In this narrative review, we aim at assessing the effect of the most relevant pathogenetic pathways related to inflammation and bone metabolism and remodeling in SpA and summarize the effects of old and new drugs on the most relevant pathogenetic pathways.

## Genetics in ax-SpA

Genetic susceptibility to axSpA is highly complex, as demonstrated by several genome-wide association studies (GWAs) [7]. Hundreds of genes, mostly immune related, have been identified to be associated with the axSpA spectrum [8], which encompasses a heterogeneous group of diseases (i.e., AS, enteropathic axSpA, and axial psoriatic arthritis) [9]. The different clinical subtypes of axSpA share a certain degree of genetics overlapping. However, significant differences do exist, suggesting a different genetic origin [10^••^]. Moreover, there is a high-shared heritability between axSpA and other often coexisting, inflammatory diseases, like psoriasis and inflammatory bowel diseases (IBDs). On the contrary, a very low degree of genetic overlapping exists between SpA and other forms of arthritis, such as rheumatoid arthritis (RA) [11]. Genetic data, in fact, indicate that nr-axSpA is etiopathogenetically more heterogeneous than AS; thus, it is possible that some nr-axSpA patients have a form of axSpA that is genetically distinct from AS [10^••^]. In this multifaceted scenario, sex-related genes and epigenetics factors also play a role in the susceptibility as well as in the clinical phenotype of axSpA and in its response to treatment [12].

The MHC system plays a fundamental role in axSpA pathogenesis, with the HLA-B27 being the strongest genetic factor of this group associated with disease susceptibility [13]. Different hypotheses have been developed to explain the role of this association in SpA pathogenesis, e.g., molecular mimicry phenomena and misfolding of the HLA-B27 heavy chain with a direct proinflammatory action through stimulation of Th17 cells [14, 15]. Recently, it has also been described that HLA-B27 could alter gut microbiota leading to an aberrant inflammatory response. Moreover, HLA-B27-positive SpA patients have a microbiota composition similar to patients with IBDs [16]. There is a higher prevalence of HLA-B27 in males compared to females, and an association between testosterone levels and HLA-B27 presence has been reported [17]. HLA-B27 contributes by 20.44% to axSpA heritability, 7.38% is due to other genes, and the rest is unexplained [9]. Studies using immunochip microarrays have identified several associations with other HLA-B alleles (e.g., HLA-B51, which is mostly associated with Behcet’s disease), while other variants have been identified in the HLA-A, HLA-DPB1, and HLA-DRB1 loci [18]. Involvement of other non-HLA MHC genes, such as MICA, TNF, TAP1, TAP2, and LMP2, has also been suggested but not confirmed because of the linkage disequilibrium with HLA-B27 [19].

More recently, the development of GWAS has resulted in the identification of additional non-MHC susceptibility loci for axSpA. Two of these loci (ERAP1 and IL23R) are of particular interest because they put under spotlight the important biological pathways involved in SpA pathogenesis that may have a potential therapeutic impact [20]. ERAP1 and ERAP2 code for enzymes that cut peptides to the ideal size for binding to MHC class 1 molecules [21]. Moreover, ERAP1 variants genetically interact with HLA-B27, being involved in the altered antigen presentation of the SpA pathogenetic model. Thus, inhibiting ERAP1 and/or ERAP2 functions could represent a novel therapeutic approach for SpA. Indeed, preliminary data showed that targeting ERAP1 by silencing it in antigen presenting cells suppressed Th17-mediated response [22^•^]. IL-23 is a proinflammatory cytokine necessary for Th17 differentiation and thus IL-17 production [23]. Several studies demonstrated the involvement of the IL23/Th17 pathway in SpA, and actually, therapies targeting IL-17 in this disease have been developed and currently successfully used [24]. On the contrary, therapeutics targeting IL-23 unexpectedly failed to demonstrate efficacy in AS [25].

Further axSpA-associated genetic pathways include other cytokines, kinases, and transcriptor factors with a key role in inflammation, such as STAT3, TYK2, JAK2, TNF, IL-1, and CARD9 [26], but additional functional studies are necessary to better elucidate how these variants contribute to susceptibility and phenotypic expression of axSpA. In the case of complex multifactorial diseases like axSpA, most DNA single-nucleotide polymorphisms (SNPs) do not cause protein coding changes, but most of these are in noncoding regions where they have epigenetic regulatory effects [8]. This could be the case for RUNX3 which encodes a transcriptor factor fundamental for CD8 lymphocyte differentiation [27]. SNPs in the region upstream the promoter of RUNX3 are strongly associated with AS, and these SNPs also showed an association with CD8+ T-cell count [28]. Moreover, Vecellio et al. identified two SNPs located upstream the promoter region of RUNX3, one affecting RUNX3 gene expression in CD8+ T cells and the other with potential regulatory functions in monocytes, suggesting that RUNX3 role in axSpA might be more complex than simply being related to reduced CD8+ T-cell count [8]. Another transcriptor factor gene associated with axSpA is TBX21, which encodes for T-bet [27]. This transcriptor factor is widely expressed in immune cells being involved in differentiation and function of CD4+ and CD8+ T cells, NK cells, and B cells. Enhanced T-bet expression in AS has been reported in both CD8+ T cells and NK cells, promoting their proinflammatory features [29].

GWAS had the potential to identify key susceptibility genes in a polygenic complex disorder like axSpA; however, since we are now approaching the post-GWAS era, the new challenge is to perform functional studies with the aim to identify the biological mechanisms behind specific genetics associations and their interplay. An integrative approach is needed to apply a real bench to bedside strategy in order to develop new therapeutics with an acceptable benefit/risk profile [8, 9]. Finally, not only genetic variants but also epigenetic mechanisms, such as DNA methylation, histone modification, and noncoding RNAs, have been shown to play an important role in axSpA scenario. For instance, alterations of histone H3 (H3K27ac and H3K4me1) seem to be correlated with RUNX3 expression [30]. Among epigenetic mechanisms, microRNAs (miRNAs) are certainly the most intriguing. They are small noncoding RNAs which can regulate gene expression. There are 14 miRNAs that are less expressed in axSpA patients compared to healthy controls, and interestingly, most of them play a role in osteoblast differentiation [31]. Finally, it has to be kept in mind that sex hormones can also influence epigenetic modulation and expression of miRNAs involved in rheumatic diseases [32].

## Innate and Acquired Immune Response in axSpA

### Innate Immune System

Evidence supports the role of innate immune system in AS pathogenesis due to its link with many AS susceptibility genes, as well as with bacteria and mechanical stress [33]. Immune system cells regulate the expression of various cytokines involved in AS pathogenesis, such as IL-1, TNF-α, IFNs, IL-17, and IL-23. TNF-α (now renamed TNF) was the first cytokine explored, and several studies underlined its role in the pathogenesis of SpA diseases [34^•^]. It is a pleiotropic cytokine produced by many cells, particularly T cells and macrophages. Evidences support the central role of TNF in AS pathogenesis. In fact, raised levels of TNF in sacroiliac joints with erosion, and high rate of the circulating soluble TNF receptors (sTNF-R1 and sTNF-R2), have been documented in AS patients, and their level correlated with disease activity. Moreover, mice which overexpressed TNF develop spinal changes, such as new bone formation [35–38]. Several investigations have shown that TNF-blocking agents are highly efficacious in controlling inflammation, improving clinical outcome and bone mineral density of AS patients, and reducing radiographic progression [39]. It is known that TNF initiates and regulates the cytokine cascade during the inflammatory response by inducing other cytokines, such as IL-1 and IL-6, which in turn recruit immune and inflammatory cells [40]. IL-1, including IL-1α and IL-1β, through their binding with its activator receptor IL-1R1, act as potent inflammatory cytokines. In animal models of arthritis, IL-1α and IL-1β seem to activate osteoclast and cause bone resorption, in addition to stimulating production of other enzymes involved in joint destruction. Further studies and genomic analysis confirmed the role of IL-1 in AS pathogenesis, although treatment with IL-1 antagonists has generally failed in AS patients [33, 41, 42].

In the last few years, IL-23/IL-17 axis has been associated with the pathogenesis of axSpA. IL-17 is a proinflammatory cytokine with many isoforms (named IL-17A, IL-17B, IL-17C, IL-17D, IL-17E, IL-17F), whereas IL-23 is a heterodimer, consisting of a p40 (shared with IL-12) and p19 chains. It has been reported that mice overexpressing IL-23 developed enthesitis and peripheral arthritis, through T-helper 17 (Th17) cells activation and production of IL-17A, IL-22, and IL-17F [43]. IL-17 has a role in inflammation and bone homeostasis and causes both synovial inflammation and joint destruction, boosting activation of NF-kB ligand (RANKL) in osteoclasts and inducing fibroblasts, osteoblasts, and chondrocytes [34^•^]. Genetic studies indicate that some loci involved in NFkB signaling, as well CARD9, can promote secretion of IL-17 and IL-23 and influence bone ossification and radiographic progression observed in AS [42]. Of note, emerging findings suggest an uncoupled action of IL-23 and IL-17 in axSpA and hypothesize a pathogenic role of IL-23 in the initiation of AS but not in maintaining established disease [44]. This theory provides a possible explanation for the negative results of trials evaluating the therapeutic role of IL-23 inhibitors in AS.

Among innate immune cells, macrophages play an important role in the AS pathogenesis by inducing production of inflammatory cytokines. Macrophages have been classified into two subpopulations: M1 (classically activated) and M2 (alternatively activated). M1 macrophages, when exposed to interferon (IFN)-γ and TNF, produce proinflammatory cytokines, such as IL-1β, TNF, IL-12, and IL-18 [45]. On the contrary, CSF-1, IL-4, IL-10, TGF-β, IL-13, fungal and helminth infections, immune complexes, and complement system favor M2 subpopulation polarization. They are involved, among others, in tissue remodeling [46]. In addition, macrophages play a role in osteoclastogenesis: CD68 macrophages have been shown in sacroiliac tissue sample of AS patients, contributing to bone resorption [35]. Moreover, misfolding of HLA-B27 induces endoplasmic reticulum (ER) stress, IL-23 production in macrophages, and immune dysregulation [47].

Histological findings from synovial biopsies of inflamed peripheral joints in SpA patients have revealed infiltration of innate immune cells, especially the CD163+ macrophages. This CD163 glycoprotein is suggested as a biologic marker for M2 macrophages and polymorphonuclear leucocytes (PMNs). Moreover, there is a positive correlation between these cells and disease activity [48]. Along with the overexpression of CD163+ cells, a decrease of M1-related cytokines was observed, indicating that M2 macrophages are the main driver of AS inflammation [49]. Of interest, a lower activation and differentiation of osteoclast, and thus a decrease of bone destruction, were detected in a study from an in vivo mouse model [50]. In this model, treatment with inhibitor of IL-4, which is the main inductor of M2 production, reduced the severity and incidence of arthritis, as well as the expression of RANK-L in macrophages [50]. It is also suggested that there is a lower expression of IFN-γ by macrophages from AS patients. This, in turn, leads to a greater Th17 response that contributes to inflammation and resultant damage in SpA [51]. Local production of IL-23 at entheseal level by HLA-B27+ macrophages has been demonstrated [52], pointing out the role of macrophages in IL-23/17 axis in pathogenesis of AS. Among the cells involved in this axis are dendritic cells (DC) that have the role in the initiation of the immune responses. A reduction in CD1c+ subset in AS patients leading to a higher expression of mononuclear cells able to induce the secretion of IL-1β and IL-6 from T cells, accompanied by Th17 immune response, has been reported [53]. Of note, DCs have been found in affected tissues of axSpA patients, implicating an immune-regulating role of these cells not only in inflammation but also in bone formation [54]. Indeed, an overexpression of ADAMTS15, which encodes for metallopeptidase and a transcription of other genes associated with WNT signaling pathway of bone formation in axSpA subjects, has been reported [55]. Other studies suggest an interaction between DC-T cell and osteoclast activation regulated by rs8092336 SNP within RANK in AS patients [56].

Natural killer (NK) cells also form a critical component of innate immunity, and they have a recognized role in AS pathogenesis. In fact, the genetic susceptibility as well as the altered expression of NK receptors (KIRs)n and HLA alleles determinate predisposition to autoimmune disorders [33]. Many studies suggested a higher level of circulating NK cells in SpA patients compared to healthy controls, as well as an increased expression of activating KIR receptors, allowing the recruitment of other immune cells [57, 58]. Accordingly, levels of NK cells are correlated with Bath AS disease activity index (BASDAI) score [59]. Furthermore, there is an increased KIR3DL2 expression on NK and T CD4+ cells in the blood and synovial mononuclear cells of SpA patients [60]. Likewise, a binding from HLA-B27 dimers and KIR3DL on cell surface has been reported [61]. These events warranted the differentiation and the expression of Th17 cells [62].

Beyond innate immune cells, other subtypes of cells participate in AS pathogenesis due to their ability to stimulate the production of IL-17, such as invariant NK T (iNKT) cells, mast cells, resident RORγt+CD3+CD4-CD8-lymphoid cells, and innate-like lymphocyte (ILC) 3 cells. iNKT cells are a specialized T-cell population that recognizes lipid antigens that are presented by CD1d, a cell-surface molecule. They have shown to have an important role in immune response, and their production is regulated by antigen-presenting cells (APCs). In mice, a small subpopulation of IL-17A-producing iNKTs which express the RORγT transcription factor IL-23R have been reported, and these cells have also been detected in humans and are involved in IL-17A and IL-22 production [63^••^]. Gamma delta (γδ) T cells are unconventional T cells, distinguish in four populations according to their function (IL-17 producers, IFNγ producers, innate-like T γδ, and γδ T regulatory cells) [63^••^]. So, these cells could produce IL-17, and they are found in large number in peripheral blood mononuclear cells (PBMCs) from AS patients, as well as in inflamed entheses where they induce IL17 production [64]. In addition, some evidence reported a maintenance of IL-17A producing cells by IL-2 [65].

ILC are tissue-resident innate immune cells involved in host defense and in tissue remodeling [63^••^]. Among ILC, the subtype 3 seems to be critical for gut permeability and interactions between microbiota and CD4+ T cells, supporting the linkage between gut inflammation and SpA pathogenesis [66]. In the last few years, researchers have focused on mucosal-associated invariant T (MAIT) cells, which express cytokines, such as IFNγ and IL-17, and whose expression is reduced in AS patients [67]. Nevertheless, their exact role in AS is still not clear. Studies suggest that stromal cells are the major effectors of the structural damage in SpA.

Stem cells are multipotent cells that include numerous cell types with various functions, like immunomodulation, differentiation in osteoblast, adipocytes, and chondroblast [68]. It is previously reported that mesenchymal stromal cells (MSCs) could inhibit T-cell proliferation, but this action in AS patients is still under investigation [69]. Some studies indicated a higher capacity of bone marrow mesenchymal stromal cells (BM-MSCs) from AS patients (AS-MSCs) to become osteoblasts through the overexpression of ERK signaling pathway and downregulation of Noggin, resulting in new bone formation [70]. Moreover, some cytokines regulate AS-MSC proliferation, migration, and function. In particular, production of IL-23 after a biomechanical stress and, consequently, IL-22 expression induced MSCs proliferation and osteogenetic differentiation, whereas FN-γ and TNFα suppressed MSCs osteogenesis [71]. It was also demonstrated that AS-MSCs produced CCL2 during osteogenesis, causing monocyte migration, macrophage polarization in proinflammatory type, and increased TNFα secretion in the enthesis [72]. MSCs from inflamed enthesis as well as from spinal ankylosing site of AS patients improved mineralization via HLA-B27-dependent activation of sXBP1/RARB axis/TNAP. In keeping with this, experiments in vitro and in vivo have shown that TNAP inhibitors blocked mineralization of MSCs and a stopped bony ankylosis. These findings further point out the importance of HLA-B27 in AS pathogenesis [73]. The pleiotropic effects of MSCs are underlined also by their capacity to regulate osteoclastogenesis. A recent study has proven a greater ability of AS-MSCs to inhibit osteoclastogenesis that seems to be mediated by an overexpression of CXCL5, which inhibits osteoclastogenesis, and it is not observed in MSCs from healthy donators [74].

### Adaptive Immune System

The role of acquired immune system in AS pathogenesis is supported by many studies. It was documented that the presence of antibodies directed against synthetic peptides from enteric bacteria that have homology sequence with HLA-B27 [75] (Note: the Ref. no. 75 is titled “Molecular mimicry of an HLA-B27-derived ligand of arthritis-linked subtypes with **chlamydial** proteins”; chlamydia do not fall in the category of enteric bacteria). Histological data on synovial biopsies of AS patients has provided evidence of B-cell-rich follicles, as well as aggregates of T cells and B cells in germinal center-like structure [76]. Moreover, the pathogenic role of IL-17 and IL-23 corroborate the involvement of T cells and adaptative immune system in AS [77]. T cells can divide in two major subtypes, CD8+ and CD4+ (also named “helper”), beyond another distinct population of T cells called “regulatory T cells” (Treg). Alteration of T lymphocytes has been found in AS, including increased percentages of T helper (Th)-1 and Th17, which also result in an imbalance of Th1/Th2 and Th17/Treg [78]. Th1 cells are a subset of CD4+ cells implicated in defense against intracellular pathogens by production of IFNγ, which acts as a macrophage-activating factor. In addition, they secrete other cytokines like TNF, IL-2, and IL-10, which are involved in inflammatory response. Conversely, Th2 cells have anti-inflammatory actions and are associated with allergic diseases, through production of IL-4, IL-5, and IL-13 [79^•^].

A skewness of Th1/Th2 was observed in AS. Th1 cells were founded in greater number in AS subject compared to healthy controls [5]. As proof, IFNγ and TNFα were higher in AS population and worse inflammatory conditions in these patients [80]. Moreover, treatment with TNF inhibitor (TNFi) drugs reduces IFNγ serum levels and the percentage of Th1 cells and is suggested to block migration of immune cells from lymph nodes to peripheral tissues [81]. Th17 cells have a key part in AS pathogenesis, being the main source of IL-17 production. Th17 differentiation is controlled by STAT-3 and STAT-5 signaling, through cytokines that could operate as activator or inhibitor factors. IL-6 and TGF-β upregulate STAT-3, leading to activation of IL-17 promoter, and on the other hand, IL-2 induces STAT-5, causing inhibition of Th17 differentiation [5]. Th17 cells were found to be increased in peripheral blood and synovium of AS patients [82]. Imbalance in IL-7 production prompts activation of fibroblasts, endothelial cells, dendritic cells, and macrophages, resulting in an inflammatory state and joint destruction [83]. Also, IL-23 play a pivotal role on expansion and maintenance of Th17 cells, and the presence of IL-23 expressing cells in human enthesis underlines a T-cell polarization at local sites [84]. Nevertheless, some recent studies have revealed that IL-17 could be produced in an IL-23-independent way. To support this hypothesis, anti-IL-23 drugs have failed in patients with axSpA, while therapies with IL-17 inhibitor improved symptoms and radiographic progression in these patients [ 63]. Moreover, Th17 cells produce IL-22, whose level has been expanded in both axial and peripheral joint tissues, as well as in mesenchymal stem cell (MSC) involved in osteogenesis. It suggests, once more, the important role of this cytokine in SpA and in new bone formation observed in this disease [71].

Treg cells are a subpopulation of T cells involved in maintenance of immune homeostasis, suppressing an excessive inflammation and autoimmune disease. Treg cells can secrete immunosuppressive cytokines such as TGF-β and IL-10, which in turn downregulates the immune response. In fact, IL-10 prevented expansion of Th17 cells and inhibited the antigen presentation [85]. Albeit their role in AS remains still controversial, findings showed an increased number of Tregs in synovial fluid of AS patients [86]. Also, it was reported an upregulation of Treg cells in PBMCs of AS patients with higher levels of ESR, CRP, and HLAB27 positivity [87]. Moreover, a Tregs convertion into Th17 cells with the above effects has also been suggested [88]. Finally, a CD56+ T-cell type with the same features of NK cells, named NKT-like cells, was uncovered. These cells seem to have both cytotoxic action and regulatory function. Some investigations reported a reduced level of NKT-like cells after IL-17A inhibition, but further studies are required to illuminate their potential pathogenic role in SpA [89^••^].

A pathogenic role of CD8+ T cells in AS has been postulated despite the evidence that HLA-B27 transgenic rats developed the disease in the absence of CD8+ cells [90]. Naïve T CD8+ cell can differentiate into effector and cytotoxic T lymphocytes (CTL) after the interaction with T-cell receptor (TCR) and mediate their pathogenic role through several different mechanisms. CD8+ effector T cells produce IFN-γ, IL-17, and TNF-α, encouraging an inflammatory milieu, while CTLs cause lysing of cells by activation of Fas/FasL pathway or secretion of perforin/granzyme. Of interest, it was demonstrated that presentation of cartilage antigens by chondrocytes to CD8+ T cells in AS subject results in cartilage destruction. In addition, CTLs could be involved in AS progression, but their exact role remains unclear and warrants further investigations [5].

Recently identified and worthy of interest, the tissue-resident memory (TRM) cells are characterized by expression of integrins and transmembrane receptors at barrier sites. After their detection in gut of patients with Crohn disease, TRM-like cells were also found in AS synovial fluid [91, 92]. Similar cells were also shown in gut, serum, and synovia of AS patients, providing an indirect support for the existence of a gut-joint axis [93]. B lymphocytes exert different roles in immune system. Besides the antibody production, these cells interact with T cells, APC, macrophages, and dendritic cells that result in production of various cytokines. Despite the little attention given to B cells over the past decades, recent investigations provide their involvement in the pathogenesis of AS [94]. Early research is shown greater levels of B cells in the serum of AS subjects compared to healthy controls and are associated with BASDAI and back pain [95]. T follicular helper (Tfh) cells can support effector B cells and induce naïve B cells to produce immunoglobulins through IL-21, an essential cytokine for B-cell proliferation and differentiation [96]. In AS patients, increased of Tfh17 cells and classic switched B cells were found, as well as their reduction after IL-17A inhibition, suggesting that B cells might take part in the pathogenesis of AS [89^••^]. Furthermore, Tfh cells may interact with B cells and facilitate the formation of germinal center and differentiation of B cells and finally the production of antibodies [79^•^].

Secretion of antibodies is boosted also by some genetic variants associated with B-cell functions, which once again emphasize the involvement of B cells in AS pathogenesis. Of these, TBX21, encoding T-bet, has been identified as a susceptibility gene for development of AS. In fact, T-bet promote Th1 differentiation and Th1 cytokine production, as well as IgM switching into IgG [27, 97, 98]. It was also suggested that T-bet+ B cells represent the germinal center-derived B cells set to become antibody-secreting cells [99]. Autoantibodies as well as the presence of B-cell infiltrates are detected in AS-affected inflammatory sites [100]. Among autoantibodies, IgG against the intracellular protein prefoldin subunit 5 (PFDN5), which is a protein with a protective role in the apoptosis of retinal cells, are recently observed in AS patients, especially in those with uveitis [101]. Besides this, antibodies directed against intracellular molecules involved in antigen presentation, like beta-2 microglobulin, have also been found in AS patients [102].

One of the most intriguing autoantibodies discovered in AS is those directed against CD74, the invariant chain of MHC class 2 that has a high affinity for the proinflammatory cytokine macrophage migration inhibitory factor (MIF). MIF, being involved in osteoclastic and osteoblastic activation, is important in radiographic progression of AS [103, 104]. As regard to bone metabolism, antibodies directed to osteoprotegerin were shown in axSpA individuals. The presence of these antibodies causes a greater expression of RANKL, leading to sustained osteoclastogenesis and, thus, bone loss [105]. Other autoantibodies against proteins that are important in the regulation of bone homeostasis, such as NAD-dependent protein deacetylase sirtuin-1 (SIRT1), sclerostin, and noggin, have been reported. SIRT1 is an intracellular enzyme that promotes osteogenesis and blocks sclerostin, an inhibitor of bone formation. The absence of SIRT1 provokes a high level of sclerostin, which means bone loss [106]. Serum IgG antibodies to SIRT1 were found mostly in early disease and in females [107]. Moreover, IgG antibodies against sclerostin and noggin, which induce bone formation, were detected in serum sera of AS patients, [108]. In AS, low serum levels of sclerostin have been correlated with formation of syndesmophytes and radiographic progression, as well as with a susceptibility to develop axSpA in patients with IBDs [109, 110]. Typically, chronic inflammation is associated with infiltration of lymphocytes, including B cells. Several studies reported an infiltration of B cells and the presence of ectopic lymphoid tissue at spine and sacroiliac joints of AS and nr-axSpA patients. Study of inflamed synovial membrane has suggested the presence of antigen-specific B memory cells that could contribute to local immune reaction. Of note, treatment with TNFi results in a reduction of neutrophils, macrophages, and T cells, but not of B cells and plasma cells. As flaring may occur shortly after the discontinuation of TNFi, it was suggested that residing B cells could be responsible for inciting relapses in AS [100]. Immune pathways and therapeutical targets are summarized in Table 1.

**Table 1:** Immune pathways and therapeutic targets

Drugs	Target	FDA-approved therapeutic indication	EMA-approved therapeutic indications	Clinical trials in axSpA	Ongoing trials in axSpA	Ref.
Etanercept	TNFα	AS PsA RA PsO	AS nr-axSpA PsA RA PsO	ASCEND ESTHER		[186, 187]
Adalimumab	TNFα	AS PsA RA PsO UC CD	AS nr-axSpA PsA RA PsO	ABILITY-1 ABILITY-3		[188, 189]
Infliximab	TNFα	AS PsA RA PsO UC CD	AS PsA RA PsO UC CD	ASSERT		[190]
Golimumab	TNFα	AS PsA RA UC	AS nr-axSpAPSA RA UC	GO-RAISE		[191–193]
Certolizumab pegol	TNFα	AS nr-axSpA PsA RA PsO CD	AS nr-axSpA PsA RA PsO CD	RAPID-axSpA ATLAS		[194, 195]
Secukinumab	IL-17A	AS nr-axSpA PsA PsO	AS nr-axSpA PsA-axPsA PsO	MAXIMISE MEASURE 1 MEASURE 2 MEASURE 3 PREVENT		[196–200]
Ixekizumab	IL-17A	AS nr-axSpA PsA PsO	AS nr-axSpA PsA PsO	COAST-V COAST-W COAST-X		[201–204]
Brodalumab	IL-17R	PsO	PsO		Phase III axSpA	[205]
Bimekizumab	IL-17A, IL-F	Not approved	PsO		Phase III AS Phase III nr-axSpA	[206–209]
Netakimab/BCD-085	IL-17R	Not approved	Not approved		Phase III AS	[210, 211]
Ustekinumab	IL-12/23	PsA PsO CD UC	PsA PsO CD UC		Phase III AS Phase III nr-axSpA	[212–214]
Tildrakizumab	IL-23	PsO	PsO		Phase II AS and nr-axSpA	[215]
Tofacitinib	JAK1/JAK3	AS PsA RA UC	AS PsA RA UC	-	Phase III AS	[216, 217]
Upadacitinib	JAK1	PsA RA UC	AS PsA RA	-	Phase III AS and nr-axSpA	[218, 219]
Filgotinib	JAK1	Not approved	RA UC	-	Phase II AS and nr-axSpA	[220, 221]
Namilumab	GM-CSF	Not approved	Not approved	-	Phase II axSpA	[222]

*FDA*, Food and Drug Administration; *EMA*, European Medicines Agency; *TNF*, tumor necrosis factor; *IL*, interleukin; *IL-17RA*, IL-17 receptor A; *JAK*, Janus kinase; *GM-CSF*, granulocyte-macrophage colony-stimulating factor; *AS*, ankylosing spondylitis; *SpA*, spondyloarthritis; *ax*, axial; *nr-ax*, non-radiographic; *PsO*, psoriasis; *PsA*, psoriatic arthritis; *RA*, rheumatoid arthritis; *CD*, Crohn’s diseases; *UC*, ulcerative colitis

## Bone Tissue

On microscopic observation, bone tissue is made up of extracellular matrix and cells. The extracellular matrix is in turn formed by an organic component consisting of type 1 collagen fibers, osteocalcin, osteonectin, glycoproteins (fibronectin, osteopontin, bone sialoprotein), proteoglycans (decorin, biglycan), an inorganic component consisting of submicroscopic of hydroxyapatite-like crystals, and calcium salts [111]. Osteoprogenitor cells (preosteoblasts) are the stem cells of bone tissue and are found on the surface of the bone trabeculae, in the connective tissue of the bone marrow cavities, and they also line the Havers and Volkmann canals. They differentiate from pluripotent mesenchymal cells and give rise to osteoblasts and other osteoprogenitor cells [112]. Osteoblasts derive from osteoprogenitor cells, and they actively participate in the formation of bone tissue by secreting the main organic components of the matrix and regulating the deposition of mineral salts. In the synthesis phase, they appear as rather large cells with a spherical nucleus, abundant basophilic cytoplasm, and alkaline phosphatase activity of its membrane [113]. Osteocytes are the most numerous cells in mature bone. They are essentially quiescent osteoblasts which, after producing bone substance, remain trapped in the calcified matrix within bone gaps. They are microscopically distinguished from osteoblasts by their flattened shape and by the numerous filiform extensions of the cytoplasm. Despite this, osteocytes are not inert cells but participate, albeit to a lesser extent than osteoblasts, in bone metabolism [114]. Osteoclasts are polynuclear giant cells with a ruffled border and a slightly acidophilic cytoplasm. They are located along the bone trabeculae within the dimples called Howship lacunae produced by their own erosive action. They are cells of monocyto-macrophage derivation; more precisely, they are syncytium derived from the fusion of these cells. They are responsible for bone resorption by mediating the acid dissolution of the inorganic extracellular matrix and enzymatically digesting the organic matrix [115^•^].

Osteogenesis begins in the embryo when clusters of embryonic connective tissue cells become more condensed and begin to express a set of genes, including Sox9 and subsequently Runx2 that encode regulatory proteins critical for the development of cartilage and bone tissue, respectively [116]. Immediately after Sox9 expression, these cells begin to proliferate and produce the cartilage matrix. They subsequently stop dividing and begin secreting Indian hedgehog (IHH) protein which causes an increase in the production of certain Wnt proteins, which activate the Wnt pathway in these cells. As a result, they deactivate Sox9 expression, retain Runx2 expression, and begin to differentiate as osteoblasts, creating a bony collar around the stem of the cartilage model [117]. Cartilage cells undergo apoptosis, leaving large cavities in the matrix, and the matrix itself becomes mineralized by the deposition of calcium phosphate crystals by osteoblasts. Osteoclasts and blood vessels invade the cavities and erode the remaining cartilage matrix, creating a space for the bone marrow, while the osteoblasts deposit trabecular bone in some parts of the cavities where fragments of cartilage matrix remain [118].

### Physiological Bone Metabolism

Contrary to what one might think, bone tissue is not permanent and immutable. Through the extracellular matrix, there are channels and cavities occupied by cells, which account for about 15% of the weight of the compact bone. These cells are engaged in a constant remodeling process, while the osteoblasts deposit new bone matrix, the osteoclasts demolish the old bone matrix, constantly renewing the bone tissue. Osteoclasts are large multinucleated cells that originate, like macrophages, from hematopoietic stem cells in the bone marrow [115^•^]. The precursor cells are released as monocytes into the bloodstream and collect at the sites of bone resorption, where they fuse to form multinucleated osteoclasts, which cling to the surfaces of the bone matrix and eat it away. Osteoclasts are able to dig deep into the substance of the compact bone, forming cavities which are then invaded by endothelial cells that form capillary structures inside [119]. The walls of the cavities formed are covered with a layer of osteoblasts that lay concentric layers of new matrix inside the cavity. Many of the osteoblasts become trapped in the bone matrix and survive by differentiating into osteocytes. The osteoblasts that make up the matrix also produce the signals that recruit and activate osteoclasts: macrophage CSF (MCSF) and TNFRSF11B (also called receptor activator of NF-κB ligand or simply RANKL). To prevent excessive matrix degradation, osteoblasts also secrete osteoprotegerin (OPG), which blocks the action of RANKL [120]. RANKL is expressed by osteoblasts, T cells, NK cells, and fibroblasts. Consequently, inflammatory cell infiltration makes a significant contribution to osteoclast formation and bone turnover [121]. The RANKL:OPG ratio determines the extent of osteoclastogenesis and is subject to various influences such as hormones, proinflammatory cytokines, biomechanical stress, and aging [122]. Several drugs can prevent osteoclastic bone loss, from estrogens to bisphosphonates to denosumab (anti-RANKL monoclonal antibodies). However, it has been noted that only few cells in vertebrae affected by AS express RANKL [123], and few SpA patients have circulating OPG antibodies [105]. The higher the level of activation of Wnt in osteoblasts, the more osteoprotegerin they secrete, and, consequently, the lower the level of activation of the osteoclasts and the lower the rate of degradation of the bone matrix. Therefore, the Wnt signaling pathway appears to have two distinct functions in bone formation; in the early stages, it controls the initial engagement of cells in the fate of osteoblasts; subsequently, it acts in the differentiated osteoblasts to help govern the balance between matrix deposition and erosion [124].

### Bone Metabolism in SpA

Similar to other chronic inflammatory diseases, including RA and IBDs, SpA is associated with systemic bone loss up to the development of osteopenia and/or osteoporosis [125]. Bone loss in SpA appears to be less pronounced than that observed in RA, also because anti-citrullinated antibodies (ACPA) induce osteoclastogenesis even in the absence of inflammation and are associated with systemic bone loss in RA before the onset of clinical disease [126]. Conversely, focal bone formation occurs in SpA and appears to be secondary to entheseal inflammation, resembling a damage response process, driven by IL-23 and IL-17. Therefore, the activation of the IL-23-IL-17 axis is associated with both catabolic and anabolic effects on bone metabolism [127^•^].

### Effects of IL-17 on Bone

IL-17 is able to regulate the activity of osteoclasts and osteoblasts. The development of bone erosions is due to the induction of osteoclastogenesis. Indeed, IL-17 directly stimulates osteoclasts by upregulating RANK (receptor for RANKL) [128] and promotes osteoclastogenesis also indirectly by inducing expression of RANKL on osteoblasts and and mesenchymal stem cells [129^••^]. In support of this concept, blockade of IL-17A by the neutralizing antibodies ixekizumab and secukinumab delayed the progression of bone erosions in patients with PsA [130]. The new entheseal bone formation results from promoting osteoblast differentiation; however, the role of IL-17 is still a matter of debate. While IL-17A is known to induce osteoblast differentiation from human mesenchymal stem cells (MSCs) [131], other studies have shown that IL-17A inhibits the differentiation of osteoblasts in mouse models, so much that using an IL-17A blocking antibody reduced bone loss [132]. Although these data are apparently contradictory, the osteoblast precursor cells that were examined in vitro in these studies were different, suggesting that the effect of IL-17A on osteoblast differentiation probably depends on the type of the cell exposed to IL-17A, the phase differentiation of that cell, and perhaps also the timing and duration of exposure to cytokines [26].

### Effects of IL-23 on Bone

Osteoblasts do not express IL-23 receptor, so IL-23 does not have any effects on the differentiation and proliferation of osteoblasts, in contrast to IL-17 [133]. On the other hand, IL-23 polarizes differentiation of the helper T (Th) cell towards Th17 cells and consequently induces IL-17 production and stimulates osteoclastogenesis [129^••^]. The direct effects of IL-23 on osteoclastogenesis are less well understood, although the induction of RANK expression has been detected on osteoclast precursor cells upon stimulation by IL-23 [134]. IL-23 could also promote osteoclastogenesis simply in the context of inflammation, and this concept is supported by limitation of progression of bone erosions in PsA patients by ustekinumab [135]. Anyway, clinical evidence shows that IL-23 blockade is less effective than inhibition of IL-17A on improving disease symptoms in the spine, suggesting potential differences between the role of IL-23 in spinal versus peripheral enthesitis [136].

### Effects of TNF on Bone

Various experimental studies evidence a link between TNF and osteoclast development; however, a direct role on osteoblast formation is currently debated [137]. Thus, clinical observations that TNFi are effective on inflammation but less so on radiological changes may be attributed to different effects on osteoclasts and osteoblasts [138]. It has been demonstrated that TNF upregulates the Wnt antagonist Dkk-1 and thereby inhibits new bone formation [139], while the blocking of Dkk-1 in experimental arthritis reversed the destructive phenotype in a remodeling pattern of peripheral and axial structural joint damage determining fusion of sacroiliac joints [140]. The potential relevance of Dkk-1 to the structural phenotype of human arthritis is indicated by low serum Dkk-1 levels in SpA [141] and the association between serum Dkk-1 levels and new bone formation in axSpA [142]. Briot and colleagues demonstrated that SpA patients receiving TNFi therapy showed a significant increase in bone mass density (BMD) in the lumbar spine over 2 years of follow-up in comparison with patients not receiving TNFi, who showed a decrease in hip BMD [143].

### Effects of IL-6 on Bone

IL-6 promotes osteoclastogenesis by inducing RANKL expression [144], while, on the other hand, IL-6 stimulates periosteal bone formation by releasing osteotransmitters from activated osteoclasts [145]. However, inhibition of IL-6 by tocilizumab and sarilumab failed to demonstrate any difference in ASAS20 response at week 12 in randomized clinical trials [146].

### Effects of GM-CSF on Bone

In 2017, Al-Mossawi and colleagues observed a significant prevalence of GM-CSF + Th17 cells in patients with SpA compared to healthy donors and RA controls and also a high percentage of cells producing GM-CSF independently of IL-17A (GM-CSF + IL-17A — cells in both CD4 and CD8 cells) [147]. The potential link between GM-CSF and bone lesions was explored in an experimental study which demonstrated that blocking GM-CSF in the SKG murine model of AS resulted in complete ablation of bone lesions, bone erosions at peripheral joints, and periosteal bone formation [148].

## Bone Metabolism Beyond SpA and Its Therapeutic Target

In SpA, there is an increased risk of clinical vertebral and nonvertebral fractures compared with healthy controls. AS was associated with an almost doubled risk of clinical vertebral fractures and a 20% increased risk of clinical nonvertebral fractures, independently of tobacco smoking, alcohol consumption, body mass index, and use of oral corticosteroids, while regular use of NSAIDs appears to eliminate the excess fracture risk related to AS, but the mechanisms involved are unknown [149]. Neumann and colleagues investigated bone geometry, microstructure, and volumetric bone mineral density (vBMD) in a cohort of patients with nr-axSpA in order to define the early bone changes occurring in axSpA and to define potential factors for deterioration of bone microstructure by using high-resolution peripheral quantitative computed tomography (HR-pQCT) at the radius. The analysis revealed, even within the first 2 years of the disease, a significantly reduced cortical area and cortical thickness in patients with nr-axSpA compared with control subjects, and moreover, total and cortical vBMD were significantly reduced in nr-axSpA patients, whereas there was no difference in trabecular vBMD [150]. The standardized method for evaluating low BMD is by dual-energy X-ray absorptiometry (DXA) which reports the standard deviation from peak bone mass (T-score) and age-matched normal values (Z-score). The World Health Organization classifies low BMD into the two categories of osteopenia and osteoporosis. Specifically, osteopenia is defined as a T-score between −1 and −2.4, and osteoporosis is defined as a T-score of less than or equal to −2.5 on DXA. The Z-scores indicate the standard deviation above or below the population normal by age, sex, weight, and ethnicity and should be used to interpret BMD in pre-menopausal women and men less than age 50. Two standard deviations below the mean are considered below the expected range [151]. Peak fracture risk has been shown to occur as early as 2.5 years after AS diagnosis, which underscores the importance of detecting and treating low BMD early in the disease course to reduce risk factors for vertebral fractures [152]. Bone metabolism mediators and effectors and therapeutic targets are summarized in Table 2.

**Table 2:** Bone metabolism and therapeutic targets

Cell	Origin	Function	Markers	Location	Targeted by	Main mechanism of action	Ref.
Osteocytes	Derived from osteoblasts after producing bone matrix	Regulate mineral composition of bone matrix	Dentin matrix protein-1, E11, sclerostin	Entrapped in bone matrix			[223]
Osteoblasts	Derived from mesenchymal precursor which also gives rise to adipocytes, chondrocytes and fibroblasts	Synthetize and mineralize bone matrix	Alkaline phosphatase, osteonectin, osteocalcin	Growing areas of bone, periosteum, endosteum	Teriparatide Romosozumab	As endogenous PTH, intermittent exposure to teriparatide will activate osteoblasts more than osteoclasts. stimulating new bone formation Inhibits sclerostin, allowing Wnt signaling in osteoblasts to promote bone formation	[224, 225]
Osteoclasts	Syncytium formed from the fusion of cells of monocyto-macrophage derivation	Bone organic and inorganic matrix resorption	Carbonic anhydrase II, calcitonin receptor, cathepsin K, vacuolar ATPase, integrin β3, RANK, cell surface aminopeptidase N/CD13	Old, unneeded, damaged areas of bone	BPS Strontium ranelate Raloxifene	NNBPS are metabolized into non-hydrolyzable analogues of ATP, inhibiting the action of ATP-dependent enzymes. NBPS inhibit farnesyl diphosphonate synthetase Binds RANKL, inhibiting osteoclast differentiation Inhibits osteoclast differentiation and promotes osteoclast apoptosis Bind ER (RLX-ER) and activate sequence of DNA (raloxifene responding element) leading to the synthesis of proteins that reduces number and activity of osteoclasts	[176, 226–228]

*RANK*, receptor activator of NF-κB; *RANKL*, receptor activator of NF-κB ligand; *BPS*, biphosphonates; *NNBPS*, non-nitrogen-containing biphosphonates; *NBPS*, biphosphonates

### Biphosphonates

Biphosphonates (BPs) are chemically stable derivatives of inorganic pyrophosphate (PPi), in which the central core P-O-P has been replaced by a non-hydrolysable P-C-P structure [153]. Since the 1960s, it is known that PPi is capable of inhibiting calcification by binding to hydroxyapatite crystals, leading to the hypothesis that regulation of PPi levels could be the mechanism by which bone mineralization is regulated [154]. Like PPi, BPs bind to hydroxyapatite crystals, and they are preferentially incorporated into sites of active bone remodeling, as commonly occurs in conditions characterized by accelerated skeletal turnover. Accordingly, BPs have become the primary therapy for skeletal disorders characterized by excessive or imbalanced skeletal remodeling, in which osteoclast and osteoblast activities are not tightly coupled, leading to excessive osteoclast-mediated bone resorption [155]. BPs used in clinical practice also have a hydroxyl group attached to the central carbon (termed the R1 position). The flanking phosphate groups provide BPs with a strong affinity for hydroxyapatite crystals in bone, whereas the hydroxyl groups further increase their ability to bind calcium, giving them their remarkable specificity for bone [156]. The presence of a nitrogen or amino group in the R2 position bound to the central carbon increases BPs antiresorptive potency by 10 to 10,000 times relative to non-nitrogen-containing BPs (NNBPs) [157]. NNBPS (etidronate, clodronate) act directly on the osteoclastic activity; in fact, they are internalized by osteoclasts and metabolized into non-hydrolyzable analogues of ATP, inhibiting the action of ATP-dependent enzymes and a cytotoxic effect with consequent cell death [158]. On the other hand, the nitrogen-containing BPs (NBPs) (pamidronate, alendronate, risedronate, zoledronate, ibandronate, neridronate) have no direct cytotoxic action, but they inhibit the enzyme farnesyl diphosphonate synthetase in the mevalonate pathway, which is necessary for the prenylation of GTP-binding proteins. This impacts on cytoskeletal proteins and intracellular trafficking, inhibiting osteoclasts actions and leading to apoptosis [159]. BPs have also anti-inflammatory or immune-modifying effects. Pamidronate, alendronate, and clodronate have been shown to inhibit the growth and differentiation of cells from bone marrow towards the monocyte/macrophage lineage [160]. The effects of BPs on cytokine generation are complex and depend on several factors, such as the cellular type, the culture assays, and the BPs class [161]. For instance, the amino BP pamidronate was shown to inhibit the production of proinflammatory cytokines in a macrophage culture system [162], and, in a model of macrophage cell-like culture, clodronate and pamidronate were shown to inhibit lipopolysaccharide-stimulated secretion of IL-1β , IL-6, and TNF [163]. In SpA, the inflammatory process is mainly localized within the subchondral bone region. In fact, a study demonstrated a high number of CD68+ macrophage and a huge expression of cathepsin K and osteoclast enzymatic activity in subchondral biopsies of AS patients compared to healthy controls [164]. Moreover, osteoporosis is a well-documented feature of SpA and can occur early [165].

All these data emerging from the early 2000s gave the rationale for the use of BPs (and especially pamidronate) in clinical trials with SpA patients. For example, Maksymowych and colleagues randomized 16 long-standing AS patients to receive treatment with pamidronate 30 mg monthly for 3 months followed by 60 mg for 3 additional months or with pamidronate 60 mg monthly for 3 months. The results showed improvement in BASDAI, bath ankylosing spondylitis functional index (BASFI), bath ankylosing spondylitis metrology index (BASMI), and erythrocyte sedimentation rate (ESR) in the group treated for 6 months [166]. Nevertheless, in other clinical trials, the same protocol regimen showed only a mild clinical improvement at month 3, with a decrease in BASDAI, but other parameters such as (BASFI, BASMI and C-reactive protein or ESR) did not change and there was a small number of ASAS 20 responders [167, 168]. Furthermore, no demographic, clinical or radiological variable predictive of response has been identified. BPs accumulate at sites of active bone turnover, and thus patients with active subchondral bone marrow inflammation in sacroiliac joints or enthesal structures evidenced by MRI may benefit from this treatment [169]. As previously mentioned, most studies with BPs in AS were carried out with pamidronate, with favorable results on symptoms and a reduction of bone marrow oedema, as detected by fat-suppressed MRI, which is considered predictive of the development of syndesmophytes in affected joints [170]. In 2014, Viapiana and colleagues compared the effect of infliximab and neridronate, a NBPS similar to pamidronate (it has been estimated that 100 mg neridronate is equivalent to 90 mg pamidronate) [171], and infliximab in patients with AS. The authors showed that neridronate was as effective as infliximab in controlling the main symptoms of AS even though, ESR and CRP remained unchanged. This might suggest that a large proportion of the symptoms in AS are unrelated to the inflammatory process per se and more likely are related to increased bone turnover at the levels of the entheses. In patients treated with neridronate a significant increase in lumbar spine BMD was observed after 6 months of therapy, while no significant changes were seen on hip sites and in patients treated with infliximab [172]. Together these observations might provide a rational for the combination of TNFi and bisphosphonates, considering the reasonable safety profile, at least over the first 5 years of treatment, and the low cost of bisphosphonates [173].

### Denosumab

RANKL is essential for osteoclast formation, function, and survival, and it is a key mediator of increased osteoclast activity in inflammatory arthritis [174]. Denosumab is a fully human monoclonal IgG2 antibody that binds and inhibits RANKL, resulting in suppression of bone resorption. Clinical studies have demonstrated that when administered subcutaneously once every 6 months, denosumab decreases bone turnover and increases bone mineral density (BMD) in postmenopausal women with low BMD [175]. Denosumab has been approved by the United States FDA for the treatment of postmenopausal women with osteoporosis at high risk for fracture, defined as a history of osteoporotic fracture, or multiple risk factors for fracture or patients who have failed or are intolerant to other available osteoporosis therapy [176]. Denosumab has also been approved for the treatment of bone loss in men receiving androgen deprivation therapy for nonmetastatic prostate cancer, for the treatment of bone loss in women receiving adjuvant aromatase inhibitor therapy for breast cancer, and for the treatment of osteoporosis due to decreased bone turnover in patients with multiple myeloma and bone metastases from breast cancer [177]. In 2008, a multicenter, randomized, double-blind, placebo-controlled, phase II study evaluated the ability of denosumab to decrease the progression of structural damage in patients with RA who were receiving methotrexate treatment. Two-hundred eighteen patients were randomized to receive denosumab 60 mg every 6 months or denosumab 180 mg every 6 months or placebo. After 12 months, the change in the MRI erosion score from baseline to 6 months was lower in the denosumab groups, and the modified Sharp erosion scores increased more with placebo treatment than with denosumab treatment [178]. This provides some evidence to support the use of denosumab to treat low BMD in AS; however, there are no studies at present of denosumab in the treatment of low BMD in AS, and thus, any potential benefit is unknown.

### Other drugs

Teriparatide is a recombinant form of human PTH, consisting of the first 34 N-terminal amino acids. Teriparatide stimulates bone formation that occurs within active remodeling sites and on surfaces of bone previously inactive. Treatment with teriparatide reduces radiographic vertebral and non-vertebral fractures compared with placebo in postmenopausal osteoporotic women [179]. Strontium ranelate inhibits osteoclast differentiation, promotes osteoclast apoptosis, activates preosteoblasts, and replaces calcium with strontium, which leads to an increase in BMD [180]. Romosozumab is a monoclonal antibody directed against sclerostin and the only available therapeutic option targeting Wnt signaling, as both bone-forming and anti-resorptive intervention to treat osteoporosis and fragility fractures [181]. Raloxifene is the only selective estrogen receptor modulator approved for long-term treatment in the prevention of osteoporotic fractures and for the reduction of invasive breast cancer risk in postmenopausal women [182]. Although these drugs may have anabolic effects on bone metabolism, there are no studies regarding their use in low BMD in SpA patients.

### bDMARDs and tsDMARDs

Better bone microstructure (higher total vBMD and trabecular vBMD, lower trabecular separation, and higher cortical thickness) and biomechanical properties (higher stiffness and failure load) were found in DMARD-treated PsA patients when compared to the no-DMARD control group. The use of bDMARD treatment was associated with better bone density and higher stiffness and failure load estimates, while no such association was observed with the use of methotrexate [183]. These observations may be explained by previous functional data showing that the two central proinflammatory mediators in PsA, IL-17, and TNF trigger an imbalance in bone homeostasis, increasing osteoclast mediated bone resorption and inhibiting osteoblast-mediated bone formation [184]. JAK inhibitors have been recently approved for the treatment of SpA. While a reduction in joint bone erosion in RA and PsA patients has been shown during treatment with tofacitinib, there are no data on BMD or osteoporosis [185].

## Conclusions

The pathogenesis of SpA is strictly related to an interplay among genetic predisposition and innate and acquired immune responses with resultant enthesitis as the *primum movens* of the disease, at both peripheral and axial levels. Furthermore, mechanical stress and mesenchymal tissue remodeling are hallmarks of SpA. Here, we reviewed the interplay of inflammatory mediators and bone metabolism. With the current knowledge of the role of bone mediators as treatment targets, making treatment approach is still a challenge in SpA management. However, encouraging findings and novelties in terms of pathogenetic mechanisms and therapeutic targets have been developed, using old and new pharmacological drugs that have a relevant impact on inflammation and bone mediators.

## References

[1] van der Linden S, Valkenburg HA, Cats A (1984). Evaluation of diagnostic criteria for ankylosing spondylitis. A proposal for modification of the New York criteria. Arthritis Rheum..

[2] Sieper J, van der Heijde D, Landewé R, Brandt J, Burgos-Vagas R, Collantes-Estevez E, Dijkmans B, Dougados M, Khan MA, Leirisalo-Repo M, van der Linden S, Maksymowych WP, Mielants H, Olivieri I, Rudwaleit M. New criteria for inflammatory back pain in patients with chronic back pain: a real patient exercise by experts from the Assessment of SpondyloArthritis international Society (ASAS). Ann Rheum Dis. 2009; 10.1136/ard.2008.101501. 10.1136/ard.2008.101501 19147614

[3] Chimenti MS, Conigliaro P, Navarini L, Martina FM, Peluso G, Birra D, Sessa P, Anzidei M, Scolieri P, Bruzzese V, Santoboni G, Cardello P, Gremese E, Afeltra A, Valesini G, Sebastiani GD, Perricone R, Scrivo R (2020). Demographic and clinical differences between ankylosing spondylitis and non-radiographic axial spondyloarthritis: results from a multicentre retrospective study in the Lazio region of Italy. Clin Exp Rheumatol..

[4] Sharip A, Kunz J. Understanding the pathogenesis of spondyloarthritis. Biomolecules. 2020; 10.3390/biom10101461. 10.3390/biom10101461 PMC7588965 33092023

[5] Rezaiemanesh A, Abdolmaleki M, Abdolmohammadi K, Aghaei H, Pakdel FD, Fatahi Y, Soleimanifar N, Zavvar M, Nicknam MH. Immune cells involved in the pathogenesis of ankylosing spondylitis. Biomed Pharmacother. 2018; 10.1016/j.biopha.2018.01.108. 10.1016/j.biopha.2018.01.108 29428668

[6] Clunie G, Horwood N. Loss and gain of bone in spondyloarthritis: what drives these opposing clinical features? Ther Adv Musculoskelet Dis. 2020; 10.1177/1759720X20969260. 10.1177/1759720X20969260 PMC7675871 33240403

[7] Australo-Anglo-American Spondyloarthritis Consortium (TASC), Reveille JD, Sims AM, Danoy P, Evans DM, Leo P, Pointon JJ, Jin R, Zhou X, Bradbury LA, Appleton LH, Davis JC, Diekman L, Doan T, Dowling A, Duan R, Duncan EL, Farrar C, Hadler J, et al. Genome-wide association study of ankylosing spondylitis identifies non-MHC susceptibility loci. Nat Genet. 2010; 10.1038/ng.513. 10.1038/ng.513 PMC3224997 20062062

[8] Vecellio M, Cohen CJ, Roberts AR, Wordsworth PB, Kenna TJ. RUNX3 and T-Bet in immunopathogenesis of ankylosing spondylitis-novel targets for therapy? Front Immunol. 2018; 10.3389/fimmu.2018.03132. 10.3389/fimmu.2018.03132 PMC6335330 30687330

[9] Costantino F, Breban M, Garchon H-J. Genetics and functional genomics of spondyloarthritis. Front Immunol. 2018; 10.3389/fimmu.2018.02933. 10.3389/fimmu.2018.02933 PMC6305624 30619293

[10.••] Brown MA, Xu H, Li Z. Genetics and the axial spondyloarthritis spectrum. Rheumatol Oxf Engl. 2020; 10.1093/rheumatology/keaa464. **Review which demonstrated that genetic variation is a major determinant of the clinical pattern of axSpA.** 10.1093/rheumatology/keaa464 PMC7566537 33053195

[11] Parkes M, Cortes A, van Heel DA, Brown MA. Genetic insights into common pathways and complex relationships among immune-mediated diseases. Nat Rev Genet. 2013; 10.1038/nrg3502. 10.1038/nrg3502 23917628

[12] Chimenti MS, Perricone C, D'Antonio A, Ferraioli M, Conigliaro P, Triggianese P, Ciccacci C, Borgiani P, Perricone R. Genetics, epigenetics, and gender impact in axial-spondyloarthritis susceptibility: an update on genetic polymorphisms and their sex related associations. Front Genet. 2021; 10.3389/fgene.2021.671976. 10.3389/fgene.2021.671976 PMC8383732 34447407

[13] Caffrey MF, James DC. Human lymphocyte antigen association in ankylosing spondylitis. Nature. 1973; 10.1038/242121a0. 10.1038/242121a0 4694299

[14] Benjamin R, Parham P. Guilt by association: HLA-B27 and ankylosing spondylitis. Immunol Today. 1990; 10.1016/0167-5699(90)90051-a. 10.1016/0167-5699(90)90051-a 2187471

[15] Colbert RA, Tran TM, Layh-Schmitt G. HLA-B27 misfolding and ankylosing spondylitis. Mol Immunol. 2014; 10.1016/j.molimm.2013.07.013. 10.1016/j.molimm.2013.07.013 PMC3857088 23993278

[16] Chimenti MS, Perricone C, Novelli L, Caso F, Costa L, Bogdanos D, Conigliaro P, Triggianese P, Ciccacci C, Borgiani P, Perricone R. Interaction between microbiome and host genetics in psoriatic arthritis. Autoimmun Rev. 2018; 10.1016/j.autrev.2018.01.002. 10.1016/j.autrev.2018.01.002 29378263

[17] Akassou A, Bakri Y. Does HLA-B27 status influence ankylosing spondylitis phenotype? Clin Med Insights Arthritis Musculoskelet Disord. 2018; 10.1177/1179544117751627. 10.1177/1179544117751627 PMC5764146 29343996

[18] Cortes A, Pulit SL, Leo PJ, Pointon JJ, Robinson PC, Weisman MH, Ward M, Gensler LS, Zhou X, Garchon HJ, Chiocchia G, Nossent J, Lie BA, Førre Ø, Tuomilehto J, Laiho K, Bradbury LA, Elewaut D, Burgos-Vargas R, et al. Major histocompatibility complex associations of ankylosing spondylitis are complex and involve further epistasis with ERAP1. Nat Commun. 2015; 10.1038/ncomms8146. 10.1038/ncomms8146 PMC4443427 25994336

[19] Reveille JD. An update on the contribution of the MHC to AS susceptibility. Clin Rheumatol. 2014; 10.1007/s10067-014-2662-7. 10.1007/s10067-014-2662-7 PMC4488903 24838411

[20] Wellcome Trust Case Control Consortium, Australo-Anglo-American Spondylitis Consortium (TASC), Burton PR, Clayton DG, Cardon LR, Craddock N, Deloukas P, Duncanson A, Kwiatkowski DP, MI MC, Ouwehand WH, Samani NJ, Todd JA, Donnelly P, Barrett JC, Davison D, Easton D, Evans DM, Leung HT, et al. Association scan of 14,500 nonsynonymous SNPs in four diseases identifies autoimmunity variants. Nat Genet. 2007; 10.1038/ng.2007.17. 10.1038/ng.2007.17 PMC2680141 17952073

[21] Saveanu L, Carroll O, Lindo V, Del Val M, Lopez D, Lepelletier Y, Greer F, Schomburg L, Fruci D, Niedermann G, van Endert PM. Concerted peptide trimming by human ERAP1 and ERAP2 aminopeptidase complexes in the endoplasmic reticulum. Nat Immunol. 2005; 10.1038/ni1208. 10.1038/ni1208 15908954

[22.•] Chen L, Ridley A, Hammitzsch A, Al-Mossawi MH, Bunting H, Georgiadis D, Chan A, Kollnberger S, Bowness P. Silencing or inhibition of endoplasmic reticulum aminopeptidase 1 (ERAP1) suppresses free heavy chain expression and Th17 responses in ankylosing spondylitis. Ann Rheum Dis. 2016; 10.1136/annrheumdis-2014-206996. **Study which demonstrated that ERAP1 inhibition may suppress Th17 response in AS**10.1136/annrheumdis-2014-206996 PMC4853590 26130142

[23] Gaffen SL, Jain R, Garg AV, Cua DJ. The IL-23-IL-17 immune axis: from mechanisms to therapeutic testing. Nat Rev Immunol. 2014; 10.1038/nri3707. 10.1038/nri3707 PMC4281037 25145755

[24] Baeten D, Sieper J, Braun J, Baraliakos X, Dougados M, Emery P, Deodhar A, Porter B, Martin R, Andersson M, Mpofu S, Richards HB; MEASURE 1 Study Group; MEASURE 2 Study Group. Secukinumab, an interleukin-17A inhibitor, in ankylosing spondylitis. N Engl J Med. 2015; 10.1056/NEJMoa1505066 10.1056/NEJMoa1505066 26699169

[25] Baeten D, Østergaard M, Wei JC, Sieper J, Järvinen P, Tam LS, Salvarani C, Kim TH, Solinger A, Datsenko Y, Pamulapati C, Visvanathan S, Hall DB, Aslanyan S, Scholl P, Padula SJ. Risankizumab, an IL-23 inhibitor, for ankylosing spondylitis: results of a randomised, double-blind, placebo-controlled, proof-of-concept, dose-finding phase 2 study. Ann Rheum Dis. 2018; 10.1136/annrheumdis-2018-213328. 10.1136/annrheumdis-2018-213328 PMC6104676 29945918

[26] Gravallese EM, Schett G. Effects of the IL-23-IL-17 pathway on bone in spondyloarthritis. Nat Rev Rheumatol. 2018; 10.1038/s41584-018-0091-8. 10.1038/s41584-018-0091-8 30266977

[27] International Genetics of Ankylosing Spondylitis Consortium (IGAS), Cortes A, Hadler J, Pointon JP, Robinson PC, Karaderi T, Leo P, Cremin K, Pryce K, Harris J, Lee S, Joo KB, Shim SC, Weisman M, Ward M, Zhou X, Garchon HJ, Chiocchia G, Nossent J, et al. Identification of multiple risk variants for ankylosing spondylitis through high-density genotyping of immune-related loci. Nat Genet. 2013; 10.1038/ng.2667. 10.1038/ng.2667 PMC3757343 23749187

[28] Ferreira MA, Mangino M, Brumme CJ, Zhao ZZ, Medland SE, Wright MJ, Nyholt DR, Gordon S, Campbell M, BP ME, Henders A, Evans DM, Lanchbury JS, Pereyra F, International HIV Controllers Study, Walker BD, Haas DW, Soranzo N, Spector TD, et al. Quantitative trait loci for CD4:CD8 lymphocyte ratio are associated with risk of type 1 diabetes and HIV-1 immune control. Am J Hum Genet. 2010; 10.1016/j.ajhg.2009.12.008. 10.1016/j.ajhg.2009.12.008 PMC2801744 20045101

[29] Lau MC, Keith P, Costello ME, Bradbury LA, Hollis KA, Thomas R, Thomas GP, Brown MA, Kenna TJ. Genetic association of ankylosing spondylitis with TBX21 influences T-bet and pro-inflammatory cytokine expression in humans and SKG mice as a model of spondyloarthritis. Ann Rheum Dis. 2017; 10.1136/annrheumdis-2015-208677. 10.1136/annrheumdis-2015-208677 27125523

[30] Cherqaoui B, Crémazy F, Hue C, Garchon H-J, Breban M, Costantino F. Epigenetics of spondyloarthritis. Joint Bone Spine. 2020; 10.1016/j.jbspin.2020.06.003. 10.1016/j.jbspin.2020.06.003 32534204

[31] Prajzlerová K, Grobelná K, Hušáková M, Forejtová Š, Jüngel A, Gay S, Vencovský J, Pavelka K, Šenolt L, Filková M. Association between circulating miRNAs and spinal involvement in patients with axial spondyloarthritis. PLoS One. 2017; 10.1371/journal.pone.0185323. 10.1371/journal.pone.0185323 PMC5609864 28938006

[32] Gracey E, Yao Y, Green B, Qaiyum Z, Baglaenko Y, Lin A, Anton A, Ayearst R, Yip P, Inman RD. Sexual dimorphism in the Th17 signature of ankylosing spondylitis. Arthritis Rheumatol. 2016; 10.1002/art.39464. 10.1002/art.39464 26473967

[33] Vanaki N, Aslani S, Jamshidi A, Mahmoudi M. Role of innate immune system in the pathogenesis of ankylosing spondylitis. Biomed Pharmacother. 2018; 10.1016/j.biopha.2018.05.097. 10.1016/j.biopha.2018.05.097 29852390

[34.•] Chimenti MS, Perricone C, Conigliaro P, Triggianese P, D’Antonio A, de Martino E, Fonti GL, Caso F, Costa L, Perricone R. Tackling the autoimmune side in spondyloarthritis: a systematic review. Autoimmun Rev. 2020; 10.1016/j.autrev.2020.102648. **Systematic review which showed that genetic background in combination with mechanical stress leads to the activation of both innate and acquired immune responses in SpA pathogenesis.** 10.1016/j.autrev.2020.102648 32801035

[35] Braun J, Bollow M, Neure L, Seipelt E, Seyrekbasan F, Herbst H, Eggens U, Distler A, Sieper J. Use of immunohistologic and in situ hybridization techniques in the examination of sacroiliac joint biopsy specimens from patients with ankylosing spondylitis. Arthritis Rheum. 1995; 10.1002/art.1780380407. 10.1002/art.1780380407 7718003

[36] Sveaas SH, Berg IJ, Provan SA, Semb AG, Olsen IC, Ueland T, Aukrust P, Vøllestad N, Hagen KB, Kvien TK, Dagfinrud H. Circulating levels of inflammatory cytokines and cytokine receptors in patients with ankylosing spondylitis: a cross-sectional comparative study. Scand J Rheumatol. 2015; 10.3109/03009742.2014.956142. 10.3109/03009742.2014.956142 25756521

[37] Christodoulou-Vafeiadou E, Geka C, Ntari L, Kranidioti K, Argyropoulou E, Meier F, Armaka M, Mourouzis I, Pantos C, Rouchota M, Loudos G, Denis MC, Karagianni N, Kollias G. Ectopic bone formation and systemic bone loss in a transmembrane TNF-driven model of human spondyloarthritis. Arthritis Res Ther. 2020; 10.1186/s13075-020-02327-4. 10.1186/s13075-020-02327-4 PMC7542121 33023659

[38] Asadbeik M, Farazmand A, Vanaki N, Mostafaei S, Jamshidi A, Ahmadzadeh N, Vojdanian M, Mohammad–Amoli M, Mahmoudi M. Gene expression profile of proinflammatory cytokines in Iranian patients with ankylosing spondylitis. Rheumatology Research. 2017; 10.22631/rr.2017.69997.1014

[39] Zambrano-Zaragoza JF, Agraz-Cibrian JM, González-Reyes C, Durán-Avelar Mde J, Vibanco-Pérez N. Ankylosing spondylitis: from cells to genes. Int J Inflam. 2013; 10.1155/2013/501653. 10.1155/2013/501653 PMC3736459 23970995

[40] Neve A, Maruotti N, Corrado A, Cantatore FP. Pathogenesis of ligaments ossification in spondyloarthritis: insights and doubts. Ann Med. 2017; 10.1080/07853890.2016.1243802. 10.1080/07853890.2016.1243802 27685190

[41] van de Loo FA, Joosten LA, van Lent PL, Arntz OJ, van den Berg WB. Role of interleukin-1, tumor necrosis factor alpha, and interleukin-6 in cartilage proteoglycan metabolism and destruction. Effect of in situ blocking in murine antigen- and zymosan-induced arthritis. Arthritis Rheum. 1995; 10.1002/art.1780380204. 10.1002/art.1780380204 7848306

[42] Chimenti MS, Sunzini F, Fiorucci L, Botti E, Fonti GL, Conigliaro P, Triggianese P, Costa L, Caso F, Giunta A, Esposito M, Bianchi L, Santucci R, Perricone R. Potential role of cytochrome c and tryptase in psoriasis and psoriatic arthritis pathogenesis: focus on resistance to apoptosis and oxidative stress. Front in Immunol. 2018; 10.3389/fimmu.2018.02363. 10.3389/fimmu.2018.02363 PMC6220124 30429845

[43] Li X, Bechara R, Zhao J, McGeachy MJ, Gaffen SL. IL-17 receptor-based signaling and implications for disease. Nat Immunol. 2019; 10.1038/s41590-019-0514-y. 10.1038/s41590-019-0514-y PMC6943935 31745337

[44] Sieper J, Poddubnyy D, Miossec P. The IL-23–IL-17 pathway as a therapeutic target in axial spondyloarthritis. Nat Rev Rheumatol. 2019; 10.1038/s41584-019-0294-7. 10.1038/s41584-019-0294-7 31551538

[45] Chávez-Galán L, Olleros ML, Vesin D, Garcia I. Much more than M1 and M2 macrophages, there are also CD169(+) and TCR(+) macrophages. Front Immunol. 2015; 10.3389/fimmu.2015.00263. 10.3389/fimmu.2015.00263 PMC4443739 26074923

[46] Martinez FO, Sica A, Mantovani A, Locati M. Macrophage activation and polarization. Front Biosci. 2008; 10.2741/2692. 10.2741/2692 17981560

[47] Akhtari M, Zargar SJ, Vojdanian M, Jamshidi A, Mahmoudi M. Monocyte-derived and M1 macrophages from ankylosing spondylitis patients released higher TNF-α and expressed more IL1B in response to BzATP than macrophages from healthy subjects. Sci Rep. 2021; 10.1038/s41598-021-96262-2. 10.1038/s41598-021-96262-2 PMC8426480 34497300

[48] Baeten D, Kruithof E, De Rycke L, Boots AM, Mielants H, Veys EM, De Keyser F. Infiltration of the synovial membrane with macrophage subsets and polymorphonuclear cells reflects global disease activity in spondyloarthropathy. Arthritis Res Ther. 2005; 10.1186/ar1501. 10.1186/ar1501 PMC1065336 15743484

[49] Vandooren B, Noordenbos T, Ambarus C, Krausz S, Cantaert T, Yeremenko N, Boumans M, Lutter R, Tak PP, Baeten D. Absence of a classically activated macrophage cytokine signature in peripheral spondylarthritis, including psoriatic arthritis. Arthritis Rheum. 2009; 10.1002/art.24406. 10.1002/art.24406 19333931

[50] Lin S, Qiu M, Chen J. IL-4 modulates macrophage polarization in ankylosing spondylitis. Cell Physiol Biochem. 2015; 10.1159/000374026. 10.1159/000374026 25896783

[51] Chu CQ, Swart D, Alcorn D, Tocker J, Elkon KB. Interferon-gamma regulates susceptibility to collagen-induced arthritis through suppression of interleukin-17. Arthritis Rheum. 2007; 10.1002/art.22453. 10.1002/art.22453 17393396

[52] Rezaiemanesh A, Mahmoudi M, Amirzargar AA, Vojdanian M, Jamshidi AR, Nicknam MH. Ankylosing spondylitis M-CSF-derived macrophages are undergoing unfolded protein response (UPR) and express higher levels of interleukin-23. Mod Rheumatol. 2017; 10.1080/14397595.2016.1259716. 10.1080/14397595.2016.1259716 27846758

[53] Wright PB, McEntegart A, McCarey D, McInnes IB, Siebert S, Milling SW. Ankylosing spondylitis patients display altered dendritic cell and T cell populations that implicate pathogenic roles for the IL-23 cytokine axis and intestinal inflammation. Rheumatology (Oxford). 2016; 10.1093/rheumatology/kev245. 10.1093/rheumatology/kev245 PMC4676904 26320138

[54] Slobodin G, Rosner I, Kessel A. Dendritic cells in the pathogenesis of ankylosing spondylitis and axial spondyloarthritis. Clin Rheumatol. 2019; 10.1007/s10067-018-4388-4. 10.1007/s10067-018-4388-4 30519775

[55] Talpin A, Costantino F, Bonilla N, Leboime A, Letourneur F, Jacques S, Dumont F, Amraoui S, Dutertre CA, Garchon HJ, Breban M, Chiocchia G. Monocyte-derived dendritic cells from HLA-B27+ axial spondyloarthritis (SpA) patients display altered functional capacity and deregulated gene expression. Arthritis Res Ther. 2014; 10.1186/s13075-014-0417-0. 10.1186/s13075-014-0417-0 PMC4292999 25142923

[56] Cortes A, Maksymowych WP, Wordsworth BP, Inman RD, Danoy P, Rahman P, Stone MA, Corr M, Gensler LS, Gladman D, Morgan A, Marzo-Ortega H, Ward MM, SPARCC (Spondyloarthritis Research Consortium of Canada), TASC (Australo-Anglo-American Spondyloarthritis Consortium), Learch TJ, Reveille JD, Brown MA, Weisman MH. Association study of genes related to bone formation and resorption and the extent of radiographic change in ankylosing spondylitis. Ann Rheum Dis. 2015; 10.1136/annrheumdis-2013-204835. 10.1136/annrheumdis-2013-204835 PMC4470170 24651623

[57] Scrivo R, Morrone S, Spadaro A, Santoni A, Valesini G. Evaluation of degranulation and cytokine production in natural killer cells from spondyloarthritis patients at single-cell level. Cytometry B Clin Cytom. 2011; 10.1002/cyto.b.20549. 10.1002/cyto.b.20549 20687202

[58] Díaz-Peña R, Vidal-Castiñeira JR, Mulero J, Sánchez A, Queiro R, López-Larrea C. Activating killer immunoglobulin-like receptors genes are associated with increased susceptibility to ankylosing spondylitis. Clin Exp Immunol. 2015; 10.1111/cei.12568. 10.1111/cei.12568 PMC4408154 25491925

[59] Akalin N, Soy M. Natural killer and natural killer t cells as a prognostic factor for rheumatoid arthritis and ankylosing spondylitis. Int Jour of Biomed Res. 2015; 10.7439/ijbr.v6i5.2004.

[60] Chan AT, Kollnberger SD, Wedderburn LR, Bowness P. Expansion and enhanced survival of natural killer cells expressing the killer immunoglobulin-like receptor KIR3DL2 in spondylarthritis. Arthritis Rheum. 2005; 10.1002/art.21395. 10.1002/art.21395 16255049

[61] Kollnberger S, Bird L, Sun MY, Retiere C, Braud VM, McMichael A, Bowness P. Cell-surface expression and immune receptor recognition of HLA-B27 homodimers. Arthritis Rheum. 2002; 10.1002/art.10605. 10.1002/art.10605 12428240

[62] Hacquard-Bouder C, Chimenti MS, Giquel B, Donnadieu E, Fert I, Schmitt A, André C, Breban M. Alteration of antigen-independent immunological synapse formation between dendritic cells and CD4+ T cells, in HLA-B27 transgenic rat: selective impairment of costimulatory molecules engagement, by mature HLA-B27. Arthritis Rheum. 2007; 10.1002/art.22572. 10.1002/art.22572 17469106

[63.••] Rosine N, Miceli-Richard C. Innate cells: the alternative source of IL-17 in axial and peripheral spondyloarthritis? Front Immunol. 2021; 10.3389/fimmu.2020.553742. **Review which described the different IL-17 mechanisms of production, suggesting a path to understand why IL-17A blocking agents are effective in axSpA in contrast to IL-23 blocking drugs.** 10.3389/fimmu.2020.553742 PMC7821711 33488572

[64] Kusuda M, Haroon N, Nakamura A. Complexity of enthesitis and new bone formation in ankylosing spondylitis: current understanding of the immunopathology and therapeutic approaches. Mod Rheumatol. 2021; 10.1093/mr/roab057. 10.1093/mr/roab057 34918137

[65] Corpuz TM, Vazquez-Lombardi R, Luong JK, Warren J, Stolp J, Christ D, King C, Brink R, Sprent J, Webster KE. IL-2 shapes the survival and plasticity of IL-17-producing γδ T cells. J Immunol. 2017; 10.4049/jimmunol.1700335. 10.4049/jimmunol.1700335 28835458

[66] Ciccia F, Guggino G, Rizzo A, Saieva L, Peralta S, Giardina A, Cannizzaro A, Sireci G, De Leo G, Alessandro R, Triolo G. Type 3 innate lymphoid cells producing IL-17 and IL-22 are expanded in the gut, in the peripheral blood, synovial fluid and bone marrow of patients with ankylosing spondylitis. Ann Rheum Dis. 2015; 10.1136/annrheumdis-2014-206323. 10.1136/annrheumdis-2014-206323 25902790

[67] Hayashi E, Chiba A, Tada K, Haga K, Kitagaichi M, Nakajima S, Kusaoi M, Sekiya F, Ogasawara M, Yamaji K, Tamura N, Takasaki Y, Miyake S. Involvement of mucosal-associated invariant T cells in ankylosing spondylitis. J Rheumatol. 2016; 10.3899/jrheum.151133. 10.3899/jrheum.151133 27370879

[68] Berthelot JM, Le Goff B, Maugars Y. Bone marrow mesenchymal stem cells in rheumatoid arthritis, spondyloarthritis, and ankylosing spondylitis: problems rather than solutions? Arthritis Res Ther. 2019; 10.1186/s13075-019-2014-8. 10.1186/s13075-019-2014-8 PMC6854713 31722720

[69] Kuca-Warnawin E, Plebańczyk M, Bonek K, Kontny E. Direct anti-proliferative effect of adipose-derived mesenchymal stem cells of ankylosing spondylitis patients on allogenic CD4+ cells. Reumatologia. 2021; 10.5114/reum.2021.103940. 10.5114/reum.2021.103940 PMC7944962 33707791

[70] Zheng G, Xie Z, Wang P, Li J, Li M, Cen S, Tang S, Liu W, Ye G, Li Y, Wang S, Wu X, Su H, Wu Y, Shen H. Enhanced osteogenic differentiation of mesenchymal stem cells in ankylosing spondylitis: a study based on a three-dimensional biomimetic environment. Cell Death Dis. 2019; 10.1038/s41419-019-1586-1. 10.1038/s41419-019-1586-1 PMC6484086 31024000

[71] El-Zayadi AA, Jones EA, Churchman SM, Baboolal TG, Cuthbert RJ, El-Jawhari JJ, Badawy AM, Alase AA, El-Sherbiny YM, McGonagle D. Interleukin-22 drives the proliferation, migration and osteogenic differentiation of mesenchymal stem cells: a novel cytokine that could contribute to new bone formation in spondyloarthropathies. Rheumatology (Oxford). 2017; 10.1093/rheumatology/kew384. 10.1093/rheumatology/kew384 27940584

[72] Xie Z, Wang P, Li J, Li Y, Wang S, Wu X, Sun S, Cen S, Su H, Deng W, Liu Z, Ouyang Y, Wu Y, Shen H. MCP1 triggers monocyte dysfunctions during abnormal osteogenic differentiation of mesenchymal stem cells in ankylosing spondylitis. J Mol Med (Berl). 2017; 10.1007/s00109-016-1489-x. 10.1007/s00109-016-1489-x 27921117

[73] Liu CH, Raj S, Chen CH, Hung KH, Chou CT, Chen IH, Chien JT, Lin IY, Yang SY, Angata T, Tsai WC, Wei JC, Tzeng IS, Hung SC, Lin KI. HLA-B27-mediated activation of TNAP phosphatase promotes pathogenic syndesmophyte formation in ankylosing spondylitis. J Clin Invest. 2019; 10.1172/JCI125212. 10.1172/JCI125212 PMC6877322 31682238

[74] Liu W, Wang P, Xie Z, Wang S, Ma M, Li J, Li M, Cen S, Tang S, Zheng G, Ye G, Wu X, Wu Y, Shen H. Abnormal inhibition of osteoclastogenesis by mesenchymal stem cells through the miR-4284/CXCL5 axis in ankylosing spondylitis. Cell Death Dis. 2019; 10.1038/s41419-019-1448-x. 10.1038/s41419-019-1448-x PMC6389901 30804325

[75] Ramos M, Alvarez I, Sesma L, Logean A, Rognan D, Lopez de Castro JA. Molecular mimicry of an HLA-B27-derived ligand of arthritis-linked subtypes with chlamydial proteins. J Biol Chem. 2002; 10.1074/jbc.m205470200. 10.1074/jbc.M205470200 12122005

[76] Revell PA, Mayston V. Histopathology of the synovial membrane of peripheral joints in ankylosing spondylitis. Ann Rheum Dis. 1982; 10.1136/ard.41.6.579. 10.1136/ard.41.6.579 PMC1000988 6756321

[77] Stoll ML. Interactions of the innate and adaptive arms of the immune system in the pathogenesis of spondyloarthritis. Clin Exp Rheumatol. 2011; PMC3266164 21269576

[78] Wang C, Liao Q, Hu Y, Zhong D. T lymphocyte subset imbalances in patients contribute to ankylosing spondylitis. Exp Ther Med. 2015; 10.3892/etm.2014.2046. 10.3892/etm.2014.2046 PMC4247318 25452811

[79.•] Liu D, Liu B, Lin C, Gu J. Imbalance of peripheral lymphocyte subsets in patients with ankylosing spondylitis: a meta-analysis. Front Immunol. 2021; 10.3389/fimmu.2021.696973. **Meta-analysis which showed that ankylosing spondylitis is a consequence of disrupted balance of both innate immune system and acquired immune system.** 10.3389/fimmu.2021.696973 PMC8291033 34295337

[80] Wang J, Zhao Q, Wang G, Yang C, Xu Y, Li Y, Yang P. Circulating levels of Th1 and Th2 chemokines in patients with ankylosing spondylitis. Cytokine. 2016; 10.1016/j.cyto.2016.01.012. 10.1016/j.cyto.2016.01.012 26827189

[81] Limón-Camacho L, Vargas-Rojas MI, Vázquez-Mellado J, Casasola-Vargas J, Moctezuma JF, Burgos-Vargas R, Llorente L. In vivo peripheral blood proinflammatory T cells in patients with ankylosing spondylitis. J Rheumatol. 2012; 10.3899/jrheum.110862. 10.3899/jrheum.110862 22337239

[82] Zhang L, Li YG, Li YH, Qi L, Liu XG, Yuan CZ, Hu NW, Ma DX, Li ZF, Yang Q, Li W, Li JM. Increased frequencies of Th22 cells as well as Th17 cells in the peripheral blood of patients with ankylosing spondylitis and rheumatoid arthritis. PLoS One. 2012; 10.1371/journal.pone.0031000. 10.1371/journal.pone.0031000 PMC3317658 22485125

[83] Jethwa H, Bowness P. The interleukin (IL)-23/IL-17 axis in ankylosing spondylitis: new advances and potentials for treatment. Clin Exp Immunol. 2016; 10.1111/cei.12670. 10.1111/cei.12670 PMC4687521 26080615

[84] Konya C, Paz Z, Apostolidis SA, Tsokos GC. Update on the role of interleukin 17 in rheumatologic autoimmune diseases. Cytokine. 2015; 10.1016/j.cyto.2015.01.003. 10.1016/j.cyto.2015.01.003 26028353

[85] Neumann C, Scheffold A, Rutz S. Functions and regulation of T cell-derived interleukin-10. Semin Immunol. 2019; 10.1016/j.smim.2019.101344. 10.1016/j.smim.2019.101344 31727465

[86] Appel H, Wu P, Scheer R, Kedor C, Sawitzki B, Thiel A, Radbruch A, Sieper J, Syrbe U. Synovial and peripheral blood CD4+FoxP3+ T cells in spondyloarthritis. J Rheumatol. 2011; 10.3899/jrheum.110377. 10.3899/jrheum.110377 21921098

[87] Zhang L, Jarvis LB, Baek HJ, Gaston JS. Regulatory IL4+CD8+ T cells in patients with ankylosing spondylitis and healthy controls. Ann Rheum Dis. 2009; 10.1136/ard.2008.088120. 10.1136/ard.2008.088120 18647857

[88.••] Watad A, Rowe H, Russell T, Zhou Q, Anderson LK, Khan A, Dunsmuir R, Loughenbury P, Borse V, Rao A, Millner PA, Bragazzi NL, Amital H, Cuhtbert R, Wittmann M, Sharif K, Kenna T, Brown MA, Newton D, et al. Normal human enthesis harbours conventional CD4+ and CD8+ T cells with regulatory features and inducible IL-17A and TNF expression. Ann Rheum Dis. 2020; 10.1136/annrheumdis-2020-217309. **Study that demonstrated the presence of CD4+ and CD8+ T cells in the human enthesis that secrete TNF and IL-17A without IL-23 stimulation.** 10.1136/annrheumdis-2020-217309 PMC7392498 32404344

[89] Jiang Y, Yang M, Zhang Y, Huang Y, Wu J, Xie Y, Wei Q, Liao Z, Gu J. Dynamics of adaptive immune cell and NK cell subsets in patients with ankylosing spondylitis after IL-17A inhibition by secukinumab. Front Pharmacol. 2021; 10.3389/fphar.2021.738316. 10.3389/fphar.2021.738316 PMC8551761 34721027

[90] Mauro D, Simone D, Bucci L, Ciccia F. Novel immune cell phenotypes in spondyloarthritis pathogenesis. Semin Immunopathol. 2021; 10.1007/s00281-021-00837-0. 10.1007/s00281-021-00837-0 PMC7990868 33569634

[91] Zundler S, Becker E, Spocinska M, Slawik M, Parga-Vidal L, Stark R, Wiendl M, Atreya R, Rath T, Leppkes M, Hildner K, López-Posadas R, Lukassen S, Ekici AB, Neufert C, Atreya I, van Gisbergen KPJM, Neurath MF. Hobit- and Blimp-1-driven CD4 + tissue-resident memory T cells control chronic intestinal inflammation. Nat Immunol. 2019; 10.1038/s41590-018-0298-5. 10.1038/s41590-018-0298-5 30692620

[92] Qaiyum Z, Gracey E, Yao Y, Inman RD. Integrin and transcriptomic profiles identify a distinctive synovial CD8+ T cell subpopulation in spondyloarthritis. Ann Rheum Dis. 2019; 10.1136/annrheumdis-2019-215349. 10.1136/annrheumdis-2019-215349 31471299

[93] Guggino G, Rizzo A, Mauro D, Macaluso F, Ciccia F. Gut-derived CD8+ tissue-resident memory T cells are expanded in the peripheral blood and synovia of SpA patients. Ann Rheum Dis. 2021; 10.1136/annrheumdis-2019-216456. 10.1136/annrheumdis-2019-216456 31628095

[94] Ge L, Wang J, Zhu BQ, Zhang ZS. Effect of abnormal activated B cells in patients with ankylosing spondylitis and its molecular mechanism. Eur Rev Med Pharmacol Sci. 2018; 10.26355/eurrev_201805_14941. 10.26355/eurrev_201805_14941 29771402

[95] Lin Q, Gu JR, Li TW, Zhang FC, Lin ZM, Liao ZT, Wei QJ, Cao SY, Li L (2009). Value of the peripheral blood B-cells subsets in patients with ankylosing spondylitis. Chin Med J (Engl)..

[96] Bautista-Caro MB, Arroyo-Villa I, Castillo-Gallego C, de Miguel E, Peiteado D, Plasencia-Rodríguez C, Villalba A, Sánchez-Mateos P, Puig-Kröger A, Martín-Mola E, Miranda-Carús ME. Decreased frequencies of circulating follicular helper T cell counterparts and plasmablasts in ankylosing spondylitis patients naïve for TNF blockers. PLoS One. 2014; 10.1371/journal.pone.0107086. 10.1371/journal.pone.0107086 PMC4159293 25203742

[97] Koch MA, Tucker-Heard G, Perdue NR, Killebrew JR, Urdahl KB, Campbell DJ. The transcription factor T-bet controls regulatory T cell homeostasis and function during type 1 inflammation. Nat Immunol. 2009; 10.1038/ni.1731. 10.1038/ni.1731 PMC2712126 19412181

[98] Peng SL, Szabo SJ, Glimcher LH. T-bet regulates IgG class switching and pathogenic autoantibody production. Proc Natl Acad Sci U S A. 2002; 10.1073/pnas.082114899. 10.1073/pnas.082114899 PMC122806 11960012

[99] Knox JJ, Myles A, Cancro MP. T-bet+ memory B cells: generation, function, and fate. Immunol Rev. 2019; 10.1111/imr.12736. 10.1111/imr.12736 PMC6626622 30874358

[100] Wilbrink R, Spoorenberg A, Verstappen GMPJ, Kroese FGM. B Cell involvement in the pathogenesis of ankylosing spondylitis. Int J Mol Sci. 2021; 10.3390/ijms222413325. 10.3390/ijms222413325 PMC8703482 34948121

[101] Kwon OC, Lee EJ, Lee JY, Youn J, Kim TH, Hong S, Lee CK, Yoo B, Robinson WH, Kim YG. Prefoldin 5 and anti-prefoldin 5 antibodies as biomarkers for uveitis in ankylosing spondylitis. Front Immunol. 2019; 10.3389/fimmu.2019.00384. 10.3389/fimmu.2019.00384 PMC6411661 30891043

[102] Curry R, Thoen J, Shelborne C, Gaudernack G, Messner R. Antibodies to and elevations of beta 2 microglobulin in the serum of ankylosing spondylitis patients. Arthritis Rheum. 1982; 10.1002/art.1780250403. 10.1002/art.1780250403 6176246

[103] Baerlecken NT, Nothdorft S, Stummvoll GH, Sieper J, Rudwaleit M, Reuter S, Matthias T, Schmidt RE, Witte T. Autoantibodies against CD74 in spondyloarthritis. Ann Rheum Dis. 2014; 10.1136/annrheumdis-2012-202208. 10.1136/annrheumdis-2012-202208 23687263

[104] Witte T, Köhler M, Georgi J, Schweikhard E, Matthias T, Baerlecken N, Hermann KG, Sieper J, Rudwaleit M, Poddubnyy D (2020). IgA antibodies against CD74 are associated with structural damage in the axial skeleton in patients with axial spondyloarthritis. Clin Exp Rheumatol..

[105] Hauser B, Zhao S, Visconti MR, Riches PL, Fraser WD, Piec I, Goodson NJ, Ralston SH. Autoantibodies to osteoprotegerin are associated with low hip bone mineral density and history of fractures in axial spondyloarthritis: a cross-sectional observational study. Calcif Tissue Int. 2017; 10.1007/s00223-017-0291-2. 10.1007/s00223-017-0291-2 PMC5587630 28534161

[106] Chen Y, Zhou F, Liu H, Li J, Che H, Shen J, Luo E. SIRT1, a promising regulator of bone homeostasis. Life Sci. 2021; 10.1016/j.lfs.2021.119041. 10.1016/j.lfs.2021.119041 33453243

[107] Hu Q, Sun Y, Li Y, Shi H, Teng J, Liu H, Cheng X, Ye J, Su Y, Yin Y, Liu M, Wang J, Yang C. Anti-SIRT1 autoantibody is elevated in ankylosing spondylitis: a potential disease biomarker. BMC Immunol. 2018; 10.1186/s12865-018-0280-x. 10.1186/s12865-018-0280-x PMC6298004 30558548

[108] Tsui FW, Tsui HW, Las Heras F, Pritzker KP, Inman RD. Serum levels of novel noggin and sclerostin-immune complexes are elevated in ankylosing spondylitis. Ann Rheum Dis. 2014; 10.1136/annrheumdis-2013-203630. 10.1136/annrheumdis-2013-203630 23894062

[109] Appel H, Ruiz-Heiland G, Listing J, Zwerina J, Herrmann M, Mueller R, Haibel H, Baraliakos X, Hempfing A, Rudwaleit M, Sieper J, Schett G. Altered skeletal expression of sclerostin and its link to radiographic progression in ankylosing spondylitis. Arthritis Rheum. 2009; 10.1002/art.24888. 10.1002/art.24888 19877044

[110] Luchetti MM, Ciccia F, Avellini C, Benfaremo D, Guggino G, Farinelli A, Ciferri M, Rossini M, Svegliati S, Spadoni T, Bolognini L, Fava G, Mosca P, Gesuita R, Skrami E, Triolo G, Gabrielli A. Sclerostin and antisclerostin antibody serum levels predict the presence of axial spondyloarthritis in patients with inflammatory bowel disease. J Rheumatol. 2018; 10.3899/jrheum.170833. 10.3899/jrheum.170833 29419466

[111] Unal M, Creecy A, Nyman JS. The role of matrix composition in the mechanical behavior of bone. Curr Osteoporos Rep. 2018; 10.1007/s11914-018-0433-0. 10.1007/s11914-018-0433-0 PMC5948175 29611037

[112] Mizoguchi T, Ono N. The diverse origin of bone-forming osteoblasts. J Bone Miner Res. 2021; 10.1002/jbmr.4410. 10.1002/jbmr.4410 PMC8338797 34213032

[113.•] Kim JM, Lin C, Stavre Z, Greenblatt MB, Shim JH. Osteoblast-osteoclast communication and bone homeostasis. Cells. 2020; 10.3390/cells9092073. **Review which showed the complex osteoblast-osteoclast communication in preserving bone homeostasis.** 10.3390/cells9092073 PMC7564526 32927921

[114] Robling AG, Bonewald LF. The osteocyte: new insights. Annu Rev Physiol. 2020; 10.1146/annurev-physiol-021119-034332. 10.1146/annurev-physiol-021119-034332 PMC8274561 32040934

[115] Sun Y, Li J, Xie X, Gu F, Sui Z, Zhang K, Yu T. Recent advances in osteoclast biological behavior. Front Cell Dev Biol. 2021; 10.3389/fcell.2021.788680. 10.3389/fcell.2021.788680 PMC8694526 34957116

[116] Ohba S. Genome-scale actions of master regulators directing skeletal development. Jpn Dent Sci Rev. 2021; 10.1016/j.jdsr.2021.10.001. 10.1016/j.jdsr.2021.10.001 PMC8556520 34745394

[117] Bi W, Deng JM, Zhang Z, Behringer RR, de Crombrugghe B. Sox9 is required for cartilage formation. Nat Genet. 1999; 10.1038/8792. 10.1038/8792 10319868

[118] Donsante S, Palmisano B, Serafini M, Robey PG, Corsi A, Riminucci M. From stem cells to bone-forming cells. Int J Mol Sci. 2021; 10.3390/ijms22083989. 10.3390/ijms22083989 PMC8070464 33924333

[119] Park-Min KH. Mechanisms involved in normal and pathological osteoclastogenesis. Cell Mol Life Sci. 2018; 10.1007/s00018-018-2817-9. 10.1007/s00018-018-2817-9 PMC9809143 29670999

[120] Boyce BF, Xing L. Biology of RANK, RANKL, and osteoprotegerin. Arthritis Res Ther. 2007; 10.1186/ar2165. 10.1186/ar2165 PMC1924516 17634140

[121] Wythe SE, Nicolaidou V, Horwood NJ. Cells of the immune system orchestrate changes in bone cell function. Calcif Tissue Int. 2014; 10.1007/s00223-013-9764-0. 10.1007/s00223-013-9764-0 23912951

[122] Ono T, Hayashi M, Sasaki F, Nakashima T. RANKL biology: bone metabolism, the immune system, and beyond. Inflamm Regen. 2020; 10.1186/s41232-019-0111-3. 10.1186/s41232-019-0111-3 PMC7006158 32047573

[123] Walsh NC, Gravallese EM. Bone remodeling in rheumatic disease: a question of balance. Immunol Rev. 2010; 10.1111/j.0105-2896.2009.00857.x. 10.1111/j.0105-2896.2009.00857.x 20193007

[124] Steinhart Z, Angers S. Wnt signaling in development and tissue homeostasis. Development (Cambridge, England). 2018; 10.1242/dev.146589. 10.1242/dev.146589 29884654

[125.•] Kocijan R, Englbrecht M, Haschka J, Simon D, Kleyer A, Finzel S, Kraus S, Resch H, Muschitz C, Engelke K, Sticherling M, Rech J, Schett G. Quantitative and qualitative changes of bone in psoriasis and psoriatic arthritis patients. J Bone Miner Res. 2015; 10.1002/jbmr.2521. **Study which demonstrated that bone loss begins early in PsA.** 10.1002/jbmr.2521 25827104

[126] Kleyer A, Finzel S, Rech J, Manger B, Krieter M, Faustini F, Araujo E, Hueber AJ, Harre U, Engelke K, Schett G. Bone loss before the clinical onset of rheumatoid arthritis in subjects with anticitrullinated protein antibodies. Ann Rheum Dis. 2014; 10.1136/annrheumdis-2012-202958. 10.1136/annrheumdis-2012-202958 23520034

[127.••] Neerinckx B, Mechanisms LR. Impact and prevention of pathological bone regeneration in spondyloarthritis. Curr Opin Rheumatol. 2017; 10.1097/BOR.0000000000000404. **Review which highlighted that activation of the IL-23-IL-17 axis is associated with both catabolic and anabolic effects on bone metabolism.** 10.1097/BOR.0000000000000404 28376064

[128] Yago T, Nanke Y, Ichikawa N, Kobashigawa T, Mogi M, Kamatani N, Kotake S. IL-17 induces osteoclastogenesis from human monocytes alone in the absence of osteoblasts, which is potently inhibited by anti-TNF-alpha antibody: a novel mechanism of osteoclastogenesis by IL-17. J Cell Biochem. 2009; 10.1002/jcb.22326. 10.1002/jcb.22326 19728295

[129] Lee Y. The role of interleukin-17 in bone metabolism and inflammatory skeletal diseases. BMB Rep. 2013; 10.5483/bmbrep.2013.46.10.141. 10.5483/BMBRep.2013.46.10.141 PMC4133834 24148767

[130] van der Heijde D, Landewé RB, Mease PJ, McInnes IB, Conaghan PG, Pricop L, Ligozio G, Richards HB, Mpofu S. Brief report: secukinumab provides significant and sustained inhibition of joint structural damage in a phase III study of active psoriatic arthritis. Arthritis Rheumatol. 2016; 10.1002/art.39685. 10.1002/art.39685 PMC5129532 27014997

[131] Osta B, Lavocat F, Eljaafari A, Miossec P. Effects of interleukin-17A on osteogenic differentiation of isolated human mesenchymal stem cells. Front Immunol. 2014; 10.3389/fimmu.2014.00425. 10.3389/fimmu.2014.00425 PMC4151036 25228904

[132] Uluçkan Ö, Jimenez M, Karbach S, Jeschke A, Graña O, Keller J, Busse B, Croxford AL, Finzel S, Koenders M, van den Berg W, Schinke T, Amling M, Waisman A, Schett G, Wagner EF. Chronic skin inflammation leads to bone loss by IL-17-mediated inhibition of Wnt signaling in osteoblasts. Sci Transl Med. 2016; 10.1126/scitranslmed.aad8996. 10.1126/scitranslmed.aad8996 27089206

[133] Zhang JR, Pang DD, Tong Q, Liu X, Su DF, Dai SM. Different modulatory effects of IL-17, IL-22, and IL-23 on osteoblast differentiation. Mediators Inflamm. 2017; 10.1155/2017/5950395. 10.1155/2017/5950395 PMC5555000 28831209

[134] Chen L, Wei XQ, Evans B, Jiang W, Aeschlimann D. IL-23 promotes osteoclast formation by up-regulation of receptor activator of NF-kappaB (RANK) expression in myeloid precursor cells. Eur J Immunol. 2008; 10.1002/eji.200838192. 10.1002/eji.200838192 18958885

[135] Kavanaugh A, Ritchlin C, Rahman P, Puig L, Gottlieb AB, Li S, Wang Y, Noonan L, Brodmerkel C, Song M, Mendelsohn AM, McInnes IB. PSUMMIT-1 and 2 Study Groups. Ustekinumab, an anti-IL-12/23 p40 monoclonal antibody, inhibits radiographic progression in patients with active psoriatic arthritis: results of an integrated analysis of radiographic data from the phase 3, multicentre, randomised, double-blind, placebo-controlled PSUMMIT-1 and PSUMMIT-2 trials. Ann Rheum Dis. 2014; 10.1136/annrheumdis-2013-204741. 10.1136/annrheumdis-2013-204741 PMC4033146 24553909

[136] Bridgewood C, Sharif K, Sherlock J, Watad A, McGonagle D. Interleukin-23 pathway at the enthesis: the emerging story of enthesitis in spondyloarthropathy. Immunol Rev. 2020; 10.1111/imr.12840. 10.1111/imr.12840 31957051

[137] Zhao B. TNF and bone remodeling. Curr Osteoporos Rep. 2017; 10.1007/s11914-017-0358-z. 10.1007/s11914-017-0358-z PMC6408950 28477234

[138] Keat A, Bennett AN, Gaffney K, Marzo-Ortega H, Sengupta R, Everiss T. Should axial spondyloarthritis without radiographic changes be treated with anti-TNF agents? Rheumatol Int. 2017; 10.1007/s00296-016-3635-8. 10.1007/s00296-016-3635-8 28035438

[139] Diarra D, Stolina M, Polzer K, Zwerina J, Ominsky MS, Dwyer D, Korb A, Smolen J, Hoffmann M, Scheinecker C, van der Heide D, Landewe R, Lacey D, Richards WG, Schett G. Dickkopf-1 is a master regulator of joint remodeling. Nat Med. 2007; 10.1038/nm1538. 10.1038/nm1538 17237793

[140] Uderhardt S, Diarra D, Katzenbeisser J, David JP, Zwerina J, Richards W, Kronke G, Schett G. Blockade of Dickkopf (DKK)-1 induces fusion of sacroiliac joints. Ann Rheum Dis. 2010; 10.1136/ard.2008.102046. 10.1136/ard.2008.102046 19304568

[141] Heiland GR, Appel H, Poddubnyy D, Zwerina J, Hueber A, Haibel H, Baraliakos X, Listing J, Rudwaleit M, Schett G, Sieper J. High level of functional dickkopf-1 predicts protection from syndesmophyte formation in patients with ankylosing spondylitis. Ann Rheum Dis. 2012; 10.1136/annrheumdis-2011-200216. 10.1136/annrheumdis-2011-200216 22186710

[142] de Rooy DP, Yeremenko NG, Wilson AG, Knevel R, Lindqvist E, Saxne T, Krabben A, Leijsma MK, Daha NA, Tsonaka S, Zhernakova A, Houwing-Duistermaat JJ, Huizinga TW, Toes RE, Baeten DL, Brouwer E, van der Helm-van Mil AH. Genetic studies on components of the Wnt signalling pathway and the severity of joint destruction in rheumatoid arthritis. Ann Rheum Dis. 2013; 10.1136/annrheumdis-2012-202184. 10.1136/annrheumdis-2012-202184 23041840

[143] Briot K, Etcheto A, Miceli-Richard C, Dougados M, Roux C. Bone loss in patients with early inflammatory back pain suggestive of spondyloarthritis: results from the prospective DESIR cohort. Rheumatology (Oxford). 2016; 10.1093/rheumatology/kev332. 10.1093/rheumatology/kev332 26361878

[144] Yeremenko N, Zwerina K, Rigter G, Pots D, Fonseca JE, Zwerina J, Schett G, Baeten D. Tumor necrosis factor and interleukin-6 differentially regulate Dkk-1 in the inflamed arthritic joint. Arthritis Rheumatol. 2015; 10.1002/art.39183. 10.1002/art.39183 25941031

[145] Johnson RW, McGregor NE, Brennan HJ, Crimeen-Irwin B, Poulton IJ, Martin TJ, Sims NA. Glycoprotein130 (Gp130)/interleukin-6 (IL-6) signalling in osteoclasts promotes bone formation in periosteal and trabecular bone. Bone. 2015; 10.1016/j.bone.2015.08.005. 10.1016/j.bone.2015.08.005 26255596

[146] Sieper J, Porter-Brown B, Thompson L, Harari O, Dougados M. Assessment of short-term symptomatic efficacy of tocilizumab in ankylosing spondylitis: results of randomised, placebo-controlled trials. Ann Rheum Dis. 2014; 10.1136/annrheumdis-2013-203559. 10.1136/annrheumdis-2013-203559 PMC3888605 23765873

[147] Al-Mossawi MH, Chen L, Fang H, Ridley A, de Wit J, Yager N, Hammitzsch A, Pulyakhina I, Fairfax BP, Simone D, Yi Y, Bandyopadhyay S, Doig K, Gundle R, Kendrick B, Powrie F, Knight JC, Bowness P. Unique transcriptome signatures and GM-CSF expression in lymphocytes from patients with spondyloarthritis. Nat Commun. 2017; 10.1038/s41467-017-01771-2. 10.1038/s41467-017-01771-2 PMC5688161 29142230

[148] Regan-Komito D, Swann JW, Demetriou P, Cohen ES, Horwood NJ, Sansom SN, Griseri T. GM-CSF drives dysregulated hematopoietic stem cell activity and pathogenic extramedullary myelopoiesis in experimental spondyloarthritis. Nat Commun. 2020; 10.1038/s41467-019-13853-4. 10.1038/s41467-019-13853-4 PMC6952438 31919358

[149] Muñoz-Ortego J, Vestergaard P, Rubio JB, Wordsworth P, Judge A, Javaid MK, Arden NK, Cooper C, Díez-Pérez A, Prieto-Alhambra D. Ankylosing spondylitis is associated with an increased risk of vertebral and nonvertebral clinical fractures: a population-based cohort study. J Bone Miner Res. 2014; 10.1002/jbmr.2217. 10.1002/jbmr.2217 24619796

[150] Neumann A, Haschka J, Kleyer A, Schuster L, Englbrecht M, Berlin A, Figueiredo CP, Simon D, Muschitz C, Kocijan R, Resch H, Rech J, Schett G. Cortical bone loss is an early feature of nonradiographic axial spondyloarthritis. Arthritis Res Ther. 2018; 10.1186/s13075-018-1620-1. 10.1186/s13075-018-1620-1 PMC6117894 30165891

[151] Lewiecki EM, Gordon CM, Baim S, Leonard MB, Bishop NJ, Bianchi ML, Kalkwarf HJ, Langman CB, Plotkin H, Rauch F, Zemel BS, Binkley N, Bilezikian JP, Kendler DL. Hans DB, Silverman S. Bone. 2008; 10.1007/s00198-008-0689-9. 10.1007/s00198-008-0689-9 18633664

[152] Prieto-Alhambra D, Muñoz-Ortego J, De Vries F, Vosse D, Arden NK, Bowness P, et al. Ankylosing spondylitis confers substantially increased risk of clinical spine fractures: a nationwide case-control study. Osteoporos Int. 2014; 10.1007/s00198-014-2939-3. 10.1007/s00198-014-2939-3 25341971

[153] Drake MT, Clarke BL, Khosla S. Bisphosphonates: mechanism of action and role in clinical practice. Mayo Clin Proc. 2008; 10.4065/83.9.1032. 10.4065/83.9.1032 PMC2667901 18775204

[154] Fleisch H, Russell RG, Straumann F. Effect of pyrophosphate on hydroxyapatite and its implications in calcium homeostasis. Nature. 1966; 10.1038/212901a0. 10.1038/212901a0 4306793

[155] Villatoro-Villar M, Kwoh CK. Bisphosphonates, bone and joint pain. Curr Osteoporos Rep. 2021; 10.1007/s11914-021-00687-7. 10.1007/s11914-021-00687-7 34215993

[156] Russell RG. Bisphosphonates: from bench to bedside. Ann N Y Acad Sci. 2006; 10.1196/annals.1346.041. 10.1196/annals.1346.041 16831938

[157] Dunford JE, Thompson K, Coxon FP, Luckman SP, Hahn FM, Poulter CD, Ebetino FH, Rogers MJ. Structure-activity relationships for inhibition of farnesyl diphosphate synthase in vitro and inhibition of bone resorption in vivo by nitrogen-containing bisphosphonates. J Pharmacol Exp Ther. 2001; 11160603

[158] Nardi A, Ventura L, Cozzi L, Tonini G. Clodronate news of efficacy in osteoporosis. Clin Cases Miner Bone Metab. 2016; 10.11138/ccmbm/2016.13.1.033. 10.11138/ccmbm/2016.13.1.033 PMC4869950 27252741

[159] Plotkin LI, Aguirre JI, Kousteni S, Manolagas SC, Bellido T. Bisphosphonates and estrogens inhibit osteocyte apoptosis via distinct molecular mechanisms downstream of extracellular signal-regulated kinase activation. J Biol Chem. 2005; 10.1074/jbc.m412817200. 10.1074/jbc.M412817200 15590626

[160] Sansoni P, Passeri G, Fagnoni F, Mohagheghpour N, Snelli G, Brianti V, Engleman EG. Inhibition of antigen-presenting cell function by alendronate in vitro. J Bone Miner Res. 1995; 10.1002/jbmr.5650101115. 10.1002/jbmr.5650101115 8592949

[161] Haibel H, Braun J, Maksymowych WP. Bisphosphonates--targeting bone in the treatment of spondyloarthritis. Clin Exp Rheumatol. 2002;20:S162-S166 12463470

[162] Maksymowych P. Walter, Bisphosphonates-anti-inflammatory properties, current medicinal chemistry - anti-inflammatory & anti-allergy agents. 2002; 10.2174/1568014024606539

[163] Pennanen N, Lapinjoki S, Urtti A, Mönkkönen J. Effect of liposomal and free bisphosphonates on the IL-1 beta, IL-6 and TNF alpha secretion from RAW 264 cells in vitro. Pharm Res. 1995; 10.1023/a:1016281608773. 10.1023/a:1016281608773 7667201

[164] Baraliakos X, Listing J, Rudwaleit M, Sieper J, Braun J. The relationship between inflammation and new bone formation in patients with ankylosing spondylitis. Arthritis Res Ther. 2008; 10.1186/ar2496. 10.1186/ar2496 PMC2592781 18761747

[165] Toussirot E, Wendling D (2000). Bone mass in ankylosing spondylitis. Clin Exp Rheumatol.

[166] Maksymowych WP, Jhangri GS, Leclercq S, Skeith K, Yan A, Russell AS (1998). An open study of pamidronate in the treatment of refractory ankylosing spondylitis. J Rheumatol..

[167] Cairns AP, Wright SA, Taggart AJ, Coward SM, Wright GD. An open study of pulse pamidronate treatment in severe ankylosing spondylitis, and its effect on biochemical markers of bone turnover. Ann Rheum Dis. 2005; 10.1136/ard.2004.022871. 10.1136/ard.2004.022871 PMC1755338 15096328

[168] Grover R, Shankar S, Aneja R, Marwaha V, Gupta R, Kumar A. Treatment of ankylosing spondylitis with pamidronate: an open label study. Ann Rheum Dis. 2006; 10.1136/ard.2005.041392. 10.1136/ard.2005.041392 PMC1798153 16611869

[169] Toussirot E, Wendling D. Antiinflammatory treatment with bisphosphonates in ankylosing spondylitis. Curr Opin Rheumatol. 2007; 10.1097/BOR.0b013e328133f57b. 10.1097/BOR.0b013e328133f57b 17551363

[170] Maksymowych WP, Chiowchanwisawakit P, Clare T, Pedersen SJ, Østergaard M, Lambert RG. Inflammatory lesions of the spine on magnetic resonance imaging predict the development of new syndesmophytes in ankylosing spondylitis: evidence of a relationship between inflammation and new bone formation. Arthritis Rheum. 2009; 10.1002/art.24132. 10.1002/art.24132 19116919

[171] Gatti D, Antoniazzi F, Prizzi R, Braga V, Rossini M, Tatò L, Viapiana O, Adami S. Intravenous neridronate in children with osteogenesis imperfecta: a randomized controlled study. J Bone Miner Res. 2005; 10.1359/JBMR.041232. 10.1359/JBMR.041232 15824848

[172] Viapiana O, Gatti D, Idolazzi L, Fracassi E, Adami S, Troplini S, Povino MR, Rossini M. Bisphosphonates vs infliximab in ankylosing spondylitis treatment. Rheumatology (Oxford). 2014; 10.1093/rheumatology/ket321. 10.1093/rheumatology/ket321 24067888

[173] Whitaker M, Guo J, Kehoe T, Benson G. Bisphosphonates for osteoporosis--where do we go from here? N Engl J Med. 2012; 10.1056/NEJMp1202619. 10.1056/NEJMp1202619 22571168

[174] Crotti TN, Smith MD, Weedon H, Ahern MJ, Findlay DM, Kraan M, Tak PP, Haynes DR. Receptor activator NF-kappaB ligand (RANKL) expression in synovial tissue from patients with rheumatoid arthritis, spondyloarthropathy, osteoarthritis, and from normal patients: semiquantitative and quantitative analysis. Ann Rheum Dis. 2002; 10.1136/ard.61.12.1047. 10.1136/ard.61.12.1047 PMC1753975 12429533

[175] McClung MR, Lewiecki EM, Cohen SB, Bolognese MA, Woodson GC, Moffett AH, Peacock M, Miller PD, Lederman SN, Chesnut CH, Lain D, Kivitz AJ, Holloway DL, Zhang C, Peterson MC, Bekker PJ. AMG 162 Bone Loss Study Group. Denosumab in postmenopausal women with low bone mineral density. N Engl J Med. 2006; 10.1056/NEJMoa044459. 10.1056/NEJMoa044459 16495394

[176] Reid IR, Billington EO. Drug therapy for osteoporosis in older adults. Lancet. 2022; 10.1016/S0140-6736(21)02646-5. 10.1016/S0140-6736(21)02646-5 35279261

[177] Body JJ, Facon T, Coleman RE, Lipton A, Geurs F, Fan M, Holloway D, Peterson MC, Bekker PJ. A study of the biological receptor activator of nuclear factor-kappaB ligand inhibitor, denosumab, in patients with multiple myeloma or bone metastases from breast cancer. Clin Cancer Res. 2006; 10.1158/1078-0432.CCR-05-1933. 10.1158/1078-0432.CCR-05-1933 16489077

[178] Cohen SB, Dore RK, Lane NE, Ory PA, Peterfy CG, Sharp JT, van der Heijde D, Zhou L, Tsuji W, Newmark R. Denosumab Rheumatoid Arthritis Study Group. Denosumab treatment effects on structural damage, bone mineral density, and bone turnover in rheumatoid arthritis: a twelve-month, multicenter, randomized, double-blind, placebo-controlled, phase II clinical trial. Arthritis Rheum. 2008; 10.1002/art.23417. 10.1002/art.23417 18438830

[179] Lindsay R, Krege JH, Marin F, Jin L, Stepan JJ. Teriparatide for osteoporosis: importance of the full course. Osteoporos Int. 2016; 10.1007/s00198-016-3534-6. 10.1007/s00198-016-3534-6 PMC4947115 26902094

[180] Cooper C, Fox KM, Borer JS. Ischaemic cardiac events and use of strontium ranelate in postmenopausal osteoporosis: a nested case-control study in the CPRD. Osteoporosis Int. 2014; 10.1007/s00198-013-2582-4. 10.1007/s00198-013-2582-4 PMC3906542 24322476

[181] Markham A. Romosozumab: first global approval. Drugs. 2019; 10.1007/s40265-019-01072-6. 10.1007/s40265-019-01072-6 30805895

[182] Gizzo S, Saccardi C, Patrelli TS, Berretta R, Capobianco G, Di Gangi S, Vacilotto A, Bertocco A, Noventa M, Ancona E, D'Antona D, Nardelli GB. Update on raloxifene: mechanism of action, clinical efficacy, adverse effects, and contraindications. Obstet Gynecol Surv. 2013; 10.1097/OGX.0b013e31828baef9. 10.1097/OGX.0b013e31828baef9 23942473

[183] Simon D, Kleyer A, Bayat S, Tascilar K, Kampylafka E, Meinderink T, Schuster L, Petrov R, Liphardt AM, Rech J, Schett G, Hueber AJ. Effect of disease-modifying anti-rheumatic drugs on bone structure and strength in psoriatic arthritis patients. Arthritis Res Ther. 2019; 10.1186/s13075-019-1938-3. 10.1186/s13075-019-1938-3 PMC6607518 31269973

[184] Adamopoulos IE, Chao CC, Geissler R, Laface D, Blumenschein W, Iwakura Y, McClanahan T, Bowman EP. Interleukin-17A upregulates receptor activator of NF-kappaB on osteoclast precursors. Arthritis Res Ther. 2010; 10.1186/ar2936. 10.1186/ar2936 PMC2875663 20167120

[185] Costa L, Del Puente A, Peluso R, Tasso M, Caso P, Chimenti MS, Sabbatino V, Girolimetto N, Benigno C, Bertolini N, Del Puente A, Perricone R, Scarpa R, Caso F. Small molecule therapy for managing moderate to severe psoriatic arthritis. Expert Opin Pharmacother. 2017; 10.1080/14656566.2017.1378343. 10.1080/14656566.2017.1378343 28891341

[186] Guillot X, Prati C, Sondag M, Wendling D. Etanercept for treating axial spondyloarthritis. Expert Opin Biol Ther. 2017; 10.1080/14712598.2017.1347156. 10.1080/14712598.2017.1347156 28682112

[187] Proft F, Weiß A, Torgutalp M, Protopopov M, Rodriguez VR, Haibel H, Behmer O, Sieper J, Poddubnyy D. Sustained clinical response and safety of etanercept in patients with early axial spondyloarthritis: 10-year results of the ESTHER trial. Ther Adv Musculoskelet Dis. 2021; 10.1177/1759720X20987700. 10.1177/1759720X20987700 PMC7970689 33796155

[188] Sieper J, van der Heijde D, Dougados M, Mease PJ, Maksymowych WP, Brown MA, Arora V, Pangan AL. Efficacy and safety of adalimumab in patients with non-radiographic axial spondyloarthritis: results of a randomised placebo-controlled trial (ABILITY-1). Ann Rheum Dis. 2013; 10.1136/annrheumdis-2012-201766. 10.1136/annrheumdis-2012-201766 PMC3664374 22772328

[189] Landewé R, Sieper J, Mease P, Inman RD, Lambert RG, Deodhar A, Marzo-Ortega H, Magrey M, Kiltz U, Wang X, Li M, Zhong S, Mostafa NM, Lertratanakul A, Pangan AL, Anderson JK. Efficacy and safety of continuing versus withdrawing adalimumab therapy in maintaining remission in patients with non-radiographic axial spondyloarthritis (ABILITY-3): a multicentre, randomised, double-blind study. Lancet. 2018; 10.1016/S0140-6736(18)31362-X. 10.1016/S0140-6736(18)31362-X 29961640

[190] van der Heijde D, Dijkmans B, Geusens P, Sieper J, DeWoody K, Williamson P, Braun J; Ankylosing spondylitis study for the evaluation of recombinant infliximab therapy study group. Efficacy and safety of infliximab in patients with ankylosing spondylitis: results of a randomized, placebo-controlled trial (ASSERT). Arthritis Rheum. 2005; 10.1002/art.20852 10.1002/art.20852 15692973

[191] Inman RD, Davis JC Jr, Heijde Dv, Diekman L, Sieper J, Kim SI, Mack M, Han J, Visvanathan S, Xu Z, Hsu B, Beutler A, Braun J. Efficacy and safety of golimumab in patients with ankylosing spondylitis: results of a randomized, double-blind, placebo-controlled, phase III trial. Arthritis Rheum. 2008; 10.1002/art.23969 10.1002/art.23969 18975305

[192] Chimenti MS, Conigliaro P, Caso F, Costa L, Ortolan A, Triggianese P, Tasso M, Fonti GL, Lorenzin MG, Perricone R, Ramonda R. Long-term effectiveness and drug survival of golimumab in patients affected by psoriatic arthritis with cutaneous involvement. Clin Rheumatol. 2022; 10.1007/s10067-021-05874-6. 10.1007/s10067-021-05874-6 PMC8724144 34410550

[193] Sieper J, van der Heijde D, Dougados M, Maksymowych WP, Scott BB, Boice JA, Berd Y, Bergman G, Curtis S, Tzontcheva A, Huyck S, Weng HH. A randomized, double-blind, placebo-controlled, sixteen-week study of subcutaneous golimumab in patients with active nonradiographic axial spondyloarthritis. Arthritis Rheumatol. 2015; 10.1002/art.39257. 10.1002/art.39257 PMC4755041 26139307

[194] Landewé R, Braun J, Deodhar A, Dougados M, Maksymowych WP, Mease PJ, Reveille JD, Rudwaleit M, van der Heijde D, Stach C, Hoepken B, Fichtner A, Coteur G, de Longueville M, Sieper J. Efficacy of certolizumab pegol on signs and symptoms of axial spondyloarthritis including ankylosing spondylitis: 24-week results of a double-blind randomised placebo-controlled phase 3 study. Ann Rheum Dis. 2014; 10.1136/annrheumdis-2013-204231. 10.1136/annrheumdis-2013-204231 PMC3888598 24013647

[195] van der Heijde D, Sieper J, Brown S, Lavie F, Panagan A (2010). Comparison of ASAS partial remission and low ASDAS as indicators of remission-like states in ankylosing spondylitis [abstract]. Arthritis Rheum..

[196] Deodhar AA, Dougados M, Baeten DL, Cheng-Chung Wei J, Geusens P, Readie A, Richards HB, Martin R, Porter B. Effect of secukinumab on patient-reported outcomes in patients with active ankylosing spondylitis: a phase III randomized trial (MEASURE 1). Arthritis Rheumatol. 2016; 10.1002/art.39805. 10.1002/art.39805 PMC5132041 27390130

[197] Braun J, Baraliakos X, Deodhar A, Poddubnyy D, Emery P, Delicha EM, Talloczy Z, Porter B. Secukinumab shows sustained efficacy and low structural progression in ankylosing spondylitis: 4-year results from the MEASURE 1 study. Rheumatology (Oxford). 2019; 10.1093/rheumatology/key375. 10.1093/rheumatology/key375 PMC6477523 30590813

[198] Chimenti MS, Fonti GL, Conigliaro P, Sunzini F, Scrivo R, Navarini L, Triggianese P, Peluso G, Scolieri P, Caccavale R, Picchianti Diamanti A, De Martino E, Salemi S, Birra D, Altobelli A, Paroli M, Bruzzese V, Laganà B, Gremese E, et al. One-year effectiveness, retention rate and safety of secukinumab in ankylosing spondylitis and psoriatic arthritis: a real-life multicenter study. Expert Opin Biol Ther. 2020; 10.1080/14712598.2020.1761957. 10.1080/14712598.2020.1761957 32401062

[199] Pavelka K, Kivitz A, Dokoupilova E, Blanco R, Maradiaga M, Tahir H, Pricop L, Andersson M, Readie A, Porter B. Efficacy, safety, and tolerability of secukinumab in patients with active ankylosing spondylitis: a randomized, double-blind phase 3 study, MEASURE 3. Arthritis Res Ther. 2017; 10.1186/s13075-017-1490-y. 10.1186/s13075-017-1490-y PMC5741872 29273067

[200] Deodhar A, Blanco R, Dokoupilová E, Hall S, Kameda H, Kivitz AJ, Poddubnyy D, van de Sande M, Wiksten AS, Porter BO, Richards HB, Haemmerle S, Braun J. Improvement of signs and symptoms of nonradiographic axial spondyloarthritis in patients treated with secukinumab: primary results of a randomized, placebo-controlled phase III study. Arthritis Rheumatol. 2021; 10.1002/art.41477. 10.1002/art.41477 PMC7839589 32770640

[201] van der Heijde D, Cheng-Chung Wei J, Dougados M, Mease P, Deodhar A, Maksymowych WP, Van den Bosch F, Sieper J, Tomita T, Landewé R, Zhao F, Krishnan E, Adams DH, Pangallo B, Carlier H. COAST-V study group. Ixekizumab, an interleukin-17A antagonist in the treatment of ankylosing spondylitis or radiographic axial spondyloarthritis in patients previously untreated with biological disease-modifying anti-rheumatic drugs (COAST-V): 16 week results of a phase 3 randomised, double-blind, active-controlled and placebo-controlled trial. Lancet. 2018; 10.1016/S0140-6736(18)31946-9. 10.1016/S0140-6736(18)31946-9 30360964

[202] Deodhar A, Poddubnyy D, Pacheco-Tena C, Salvarani C, Lespessailles E, Rahman P, Järvinen P, Sanchez-Burson J, Gaffney K, Lee EB, Krishnan E, Santisteban S, Li X, Zhao F, Carlier H, Reveille JD. COAST-W Study Group. Efficacy and safety of ixekizumab in the treatment of radiographic axial spondyloarthritis: sixteen-week results from a phase III randomized, double-blind, placebo-controlled trial in patients with prior inadequate response to or intolerance of tumor necrosis factor inhibitors. Arthritis Rheumatol. 2019; 10.1002/art.40753. 10.1002/art.40753 PMC6593790 30343531

[203] Dougados M, Wei JC, Landewé R, Sieper J, Baraliakos X, Van den Bosch F, Maksymowych WP, Ermann J, Walsh JA, Tomita T, Deodhar A, van der Heijde D, Li X, Zhao F, Bertram CC, Gallo G, Carlier H, Gensler LS. COAST-V and COAST-W Study Groups. Efficacy and safety of ixekizumab through 52 weeks in two phase 3, randomised, controlled clinical trials in patients with active radiographic axial spondyloarthritis (COAST-V and COAST-W). Ann Rheum Dis. 2020; 10.1136/annrheumdis-2019-216118. 10.1136/annrheumdis-2019-216118 PMC7025731 31685553

[204] Deodhar A, van der Heijde D, Gensler LS, Kim TH, Maksymowych WP, Østergaard M, Poddubnyy D, Marzo-Ortega H, Bessette L, Tomita T, Leung A, Hojnik M, Gallo G, Li X, Adams D, Carlier H, Sieper J, COAST-X Study Group. Ixekizumab for patients with non-radiographic axial spondyloarthritis (COAST-X): a randomised, placebo-controlled trial. Lancet. 2020; 10.1016/S0140-6736(19)32971-X. 10.1016/S0140-6736(19)32971-X 31813637

[205] National Library of Medicine (U.S.). (2017 March – 2019 September) A phase 3 multicenter, randomized, double-blind, placebo-controlled study with an open label extension to evaluate the efficacy and safety of KHK4827 in Subjects with axial Spondyloarthritis Identifier: NCT02985983. https://clinicaltrials.gov/ct2/show/NCT02985983.

[206] Ritchlin CT, Kavanaugh A, Merola JF, Schett G, Scher JU, Warren RB, Assudani D, Kumke T, Ink B, McInnes IB. Dual neutralization of IL-17A and IL-17F with bimekizumab in patients with active Psa: results from a 48-week phase 2b, randomized, double-blind, placebo-controlled, dose-ranging study [abstract]. Arthritis. Rheumatol. 2018;70(suppl 10) https://acrabstracts.org/abstract/dual-neutralization-of-il-17a-and-il-17f-with-bimekizumab-in-patients-with-active-psa-results-from-a-48-week-phase-2b-randomized-double%e2%80%91blind-placebo-controlled-dose-ranging-study/

[207] National Library of Medicine (U.S.). (2019 April – 2022 September) A Phase 3, multicenter, randomized, double-blind, placebo-controlled study evaluating the efficacy and safety of Bimekizumab in subjects with active Aylosing Spondylitis Identifier: NCT03928743. https://www.clinicaltrials.gov/ct2/show/NCT03928743.

[208] National Library of Medicine (U.S.). (2019 April – ongoing) A phase 3, multicenter, randomized, double-blind, placebo-controlled study evaluating the efficacy and safety of Bimekizumab in subjects with active nonradiographic Axial Spondyloarthritis Identifier: NCT03928704. https://www.clinicaltrials.gov/ct2/show/NCT03928704.

[209] National Library of Medicine (U.S.). (2020 June - ongoing) A multicenter, open-label extension study to asses the long-term safety, tolerability, and efficacy of bimekizumab in the treatment of study participants with active axial Spondyloarthritis, Ankylosing Spondylitis, and Nonradiographic Axial Spondyloarthritis. Identifier: NCT04436640. https://clinicaltrials.gov/ct2/show/NCT04436640.

[210] OP0028 Efficacy and safety of bcd-085, a novel il-17 inhibitor, in ankylosing spondylitis. results of phase 2 clinical study. Ann Rheum Dis. 2018; 10.1136/annrheumdis-2018-eular.2380.

[211] ClinicalTrials.gov Identifier: NCT03447704 (n.d.)

[212] ClinicalTrials.gov Identifier: NCT02437162 (n.d.)

[213] ClinicalTrials.gov Identifier: NCT02438787 (n.d.)

[214] ClinicalTrials.gov Identifier: NCT02407223 (n.d.)

[215] ClinicalTrials.gov Identifier: NCT02980705 (n.d.)

[216] van der Heijde D, Deodhar A, Wei JC, Drescher E, Fleishaker D, Hendrikx T, Li D, Menon S, Kanik KS. Tofacitinib in patients with ankylosing spondylitis: a phase II, 16-week, randomised, placebo-controlled, dose-ranging study. Ann Rheum Dis. 2017; 10.1136/annrheumdis-2016-210322. 10.1136/annrheumdis-2016-210322 PMC5738601 28130206

[217] National Library of Medicine (U.S.). (2017 September– 2018 December) safety and efficacy of tofacitinib in the treatment of NSAID Refractory Axial Spondyloarthritis: A clinical trial. Identifier: NCT03738956. https://clinicaltrials.gov/ct2/show/NCT03738956.

[218] van der Heijde D, Song IH, Pangan AL, Deodhar A, van den Bosch F, Maksymowych WP, Kim TH, Kishimoto M, Everding A, Sui Y, Wang X, Chu AD, Sieper J. Efficacy and safety of upadacitinib in patients with active ankylosing spondylitis (SELECT-AXIS 1): a multicentre, randomised, double-blind, placebo-controlled, phase 2/3 trial. Lancet. 2019; 10.1016/S0140-6736(19)32534-6. 10.1016/S0140-6736(19)32534-6 31732180

[219] National Library of Medicine (U.S.). (2019 November – ongoing) A phase 3 randomized, placebo-controlled, double-blind program to evaluate efficacy and safety of Upadacitinib in adult subjects with Axial Spondyloarthritis followed by a Remission-Withdrawal Period Identifier: NCT04169373. https://clinicaltrials.gov/ct2/show/NCT04169373.

[220] van der Heijde D, Baraliakos X, Gensler LS, Maksymowych WP, Tseluyko V, Nadashkevich O, Abi-Saab W, Tasset C, Meuleners L, Besuyen R, Hendrikx T, Mozaffarian N, Liu K, Greer JM, Deodhar A, Landewé R. Efficacy and safety of filgotinib, a selective Janus kinase 1 inhibitor, in patients with active ankylosing spondylitis (TORTUGA): results from a randomised, placebo-controlled, phase 2 trial. Lancet. 2018; 10.1016/S0140-6736(18)32463-2. 10.1016/S0140-6736(18)32463-2 30360970

[221] National Library of Medicine (U.S.). (2019 May- ongoing) A randomized, double-blind, placebo-controlled phase 2 study to evaluate the effect of Filgotinib on semen parameters in adult males with Active Rheumatoid Arthritis, Psoriatic Arthritis, Ankylosing Spondylitis or Non-radiographic Axial Spondyloarthritis. Identifier: NCT03926195. https://clinicaltrials.gov/ct2/show/NCT03926195.

[222] National Library of Medicine (U.S.). (2018 September – 2020 February) A phase 2a study to evaluate the safety and efficacy of Namilumab in subjects with moderate-to-severely active Axial Spondyloarthritis. Identifier: NCT03622658. https://clinicaltrials.gov/ct2/show/NCT03622658.

[223] Bonewald LF. Osteocytes as dynamic multifunctional cells. Ann N Y Acad Sci. 2007; 10.1196/annals.1402.018. 10.1196/annals.1402.018 17646259

[224] Vilaca T, Eastell R, Schini M. Osteoporosis in men. Lancet Diabetes Endocrinol. 2022; 10.1016/S2213-8587(22)00012-2. 10.1016/S2213-8587(22)00012-2 35247315

[225] Aditya S, Rattan A. Sclerostin inhibition: a novel target for the treatment of postmenopausal osteoporosis. J Midlife Health. 2021; 10.4103/jmh.JMH_106_20. 10.4103/jmh.JMH_106_20 PMC8849148 35264832

[226] Peris P, Monegal A, Guañabens N. Bisphosphonates in inflammatory rheumatic diseases. Bone. 2021; 10.1016/j.bone.2021.115887. 10.1016/j.bone.2021.115887 33592328

[227] Kołodziejska B, Stępień N, Kolmas J. The influence of strontium on bone tissue metabolism and its application in osteoporosis treatment. Int J Mol Sci. 2021; 10.3390/ijms22126564. 10.3390/ijms22126564 PMC8235140 34207344

[228] Elbers LPB, Raterman HG, Lems WF. Bone mineral density loss and fracture risk after discontinuation of anti-osteoporotic drug treatment: a narrative review. Drugs. 2021; 10.1007/s40265-021-01587-x. 10.1007/s40265-021-01587-x PMC8519894 34524681

